# Anti-eczema potential of three tea extracts: mechanisms of anti-inflammatory, antibacterial, antioxidant, and immunomodulatory effects

**DOI:** 10.3389/fphar.2025.1595573

**Published:** 2025-09-22

**Authors:** Zeting Huang, Lanyue Zhang, Jie Xuan, Duoling Xu, Jiyu Weng, Bing Yu, Weihua Peng

**Affiliations:** ^1^ Guangzhou Zhongzhuang Cosmetics Co., LTD, Guangzhou, China; ^2^ School of Biomedical and Pharmaceutical Sciences, Guangdong Provincial Key Laboratory of Plant Re-sources Biorefinery, Guangdong University of Technology, Guangzhou, China; ^3^ Hospital of Stomatology, Guanghua School of Stomatology, Sun Yat-sen University, Guangzhou, China; ^4^ Department of Biochemistry and Molecular Biology, Bloomberg School of Public Health, Johns Hopkins University, Baltimore, United States; ^5^ School of Materials and Energy, Guangdong University of Technology, Guangzhou, China

**Keywords:** anti-eczema, anti-inflammatory effects, inflammatory response, pro-inflammatory cytokines, tea extracts

## Abstract

**Background:**

Tea, with a long history in China, is known for its anti-inflammatory and antioxidant properties. Limited research exists on its use in eczema treatment. This study explores the effects and mechanisms of three tea extracts—*Camellia sinensis var. assamica (Royle ex Hook.) (Theaceae)* (CS), *Camellia ptilophylla Hung T. Chang (Theaceae)* (CP), *Camellia arborescens Hung T. Chang, F. L. Yu & P. S. Wang (Theaceae)* (CA). On eczema induced by 1-chloro-2,4-dinitrobenzene (DNCB) in mice and to explore the underlying anti-inflammatory and immune regulatory mechanisms.

**Methods:**

The metabolites of tea extracts were analyzed using ultra-high performance liquid chromatography/quadrupole Orbitrap mass spectrometry (UHPLC-Q-Orbitrap-MS). A DNCB-induced dermatitis model in mice was established, with histological staining and immunohistochemistry to assess eczema lesions and cytokine expression. *In vitro* tests were performed on RAW 264.7 cells and HaCaT cells to analyze the effects on inflammation, immune regulation, and cell migration.

**Results:**

All three tea extracts alleviated DNCB-induced epidermal thickening, reduced mast cell infiltration, and decreased TNF-α and IL-1β levels. The extracts suppressed nitric oxide (NO), reactive oxygen species (ROS), and NF-κB expression. Additionally, they downregulated immune-related factors such as IL-1, IL-6, IFN-γ, and TGF-β. Scanning electron microscopy revealed morphological changes in *Escherichia coli* and *Staphylococcus aureus*.

**Conclusion:**

CS, CP, and CA show potential for treating eczema, demonstrating anti-inflammatory and immune-regulatory effects. These tea extracts could serve as promising natural treatments for eczema and related inflammatory skin conditions.

## Graphical Abstract

**Figure:**
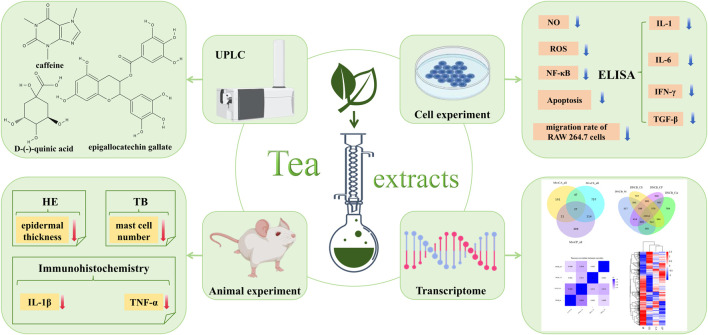

## 1 Introduction

Eczema is a complex allergic condition with multifactorial pathogenesis, progressively developing to significantly affect physical and mental health (David Boothe et al., 2017; Faraz et al., 2020). Its global adult incidence has reached 10%–20% (Liu et al., 2022), presenting symptoms such as papules, pruritus, erosion, and exudation, triggered by external factors such as immune decline, UVB exposure, and chemical irritants (Maslin et al., 2020). Current treatments rely heavily on synthetic anti-inflammatory drugs, which only provide short-term relief and carry risks of side effects or dependence–driving interest in natural botanical alternatives as safer, milder options. For example, Khalighi et al. (2021) showed that Althaea officinalis extract liposomes matched steroid efficacy in atopic eczema without significant side effects, although their study focused on clinical outcomes rather than underlying mechanisms, highlighting the need for deeper mechanistic exploration of botanical therapies. A key hallmark of eczema pathogenesis is dysregulated immune signaling, particularly cytokine imbalances and hyperactive inflammatory pathways (Sushama et al., 2019; Yang et al., 2017). Pathogenic progression is linked to elevated pro-inflammatory cytokines (IL-1, IL-6) and reduced anti-inflammatory mediators (IFN-γ) (Peters and Peters 2019; Kim et al., 2020; Meng et al., 2021), while cytokines like TNF-α and TGF-β further amplify skin inflammation by activating immune cells and disrupting barrier function (Ge et al., 2018; Jahandideh et al., 2020). These imbalances are driven by overactivation of critical signaling pathways, including NF-κB (a central regulator of inflammation) and excessive reactive oxygen species (ROS), which collectively exacerbate tissue damage and itching in eczema.

Notably, tea–with its millennia-old history of medicinal use–contains bioactive metabolites, especially catechins, that target these exact pathogenic mechanisms. Rich in polyphenolic compounds like epigallocatechin gallate (EGCG), epicatechin gallate (ECG), and epigallocatechin (EGC), tea phytochemicals exert therapeutic effects by modulating the very pathways disrupted in eczema: they suppress NF-κB activation, scavenge excess ROS, and restore cytokine balance (Alnaimat et al., 2021; Xiao and Jiang, 2015; Zhang et al., 2019; Xue et al., 2021). For example, EGCG directly inhibits mast cell degranulation (a key step in allergic responses) by reducing histamine release, while lowering Th2 cytokines (IL-4, IL-5) that drive allergic inflammation in eczema—addressing both immune cell hyperactivity and cytokine dysregulation (Zhang et al., 2019). Such multifaceted actions explain why tea extracts alleviate eczema symptoms (erythema, pruritus, lichenification) through combined anti-inflammatory, anti-edematous, and anti-pruritic effects (Xiao and Jiang, 2015). Our prior research supports this mechanistic potential: five Camellia extracts inhibited histamine-induced allergic dermatitis by reducing epidermal thickening, mast cell infiltration, and pro-inflammatory markers (IL-1β, NGF), while suppressing NF-κB in LPS-induced RAW 264.7 cells and scavenging ROS (Huang et al., 2024). These extracts also modulated mast cell degranulation and Th2 cytokine secretion, reinforcing their eczema-related activity (Sehra et al., 2016). Additionally, ethanol extracts of black, green, and white teas alleviated psoriasis-like lesions in mice by reducing pro-inflammatory cytokines (IL-17, TNF-α) and regulating inflammatory pathways—further confirming tea’s broad anti-inflammatory and antioxidant properties (Zhang et al., 2024).

This study aims to investigate the therapeutic potential and underlying mechanisms of three distinct tea extracts—*Camellia sinensis var. assamica (Royle ex Hook.) (Theaceae) (CS), Camellia ptilophylla Hung T. Chang (Theaceae) (CP), Camellia arborescens Hung T. Chang, F. L. Yu & P. S. Wang (Theaceae) (CA)* in eczematous mice induced by 1-chloro-2,4-dinitrobenzene (DNCB). The selection of these specific species is driven by their distinct bioactive metabolite profiles, which provide a rationale for comparative analysis of their therapeutic efficacy: whereas CS and CA share common metabolites such as caffeine and D-()-quinic acid, CP differs significantly by accumulating higher levels of D-()-quinic acid, (−)-gallocatechin, L-norleucine, and epigallocatechin gallate (EGCG). Specifically, CS and CA contain caffeine at levels above 20%, while CP is characterized by a high content of D-()-quinic acid (>25%) and EGCG (>10%) – differences that are critical to understanding their varying potential in eczema treatment. To elucidate their anti-inflammatory, antioxidant, and anti-photoaging properties, we evaluate the effects of these extracts on LPS-induced RAW 264.7 macrophages and UVB-induced HaCaT keratinocytes, complemented by ultra-high-performance liquid chromatography/quadrupole Orbitrap mass spectrometry (UHPLC-Q-Orbitrap-MS) to identify key bioactive metabolites. *In vivo*, DNCB-induced dermatitis models, histological staining (HE, TB), and immunohistochemistry (TNF-α, IL-1β) are used to assess lesion alleviation. *In vitro* assays include MTT for cell viability, LPS-induced inflammation models, ELISA (IL-1, IL-6, IFN-γ, TGF-β), and flow cytometry (HaCaT apoptosis/ROS), alongside scanning electron microscopy (SEM) for antimicrobial activity and transcriptomic analysis to explore molecular mechanisms. This work aims to provide a scientific basis for the development of CS, CP, and CA as natural agents for eczema and related inflammatory skin conditions.

## 2 Materials and methods

### 2.1 Materials

*Camellia sinensis var. assamica (Royle ex Hook.) (Theaceae)* (CS)*, Camellia ptilophylla Hung T. Chang (Theaceae)* (CP)*, Camellia arborescens Hung T. Chang, F. L. Yu & P. S. Wang (Theaceae)* (CA) were collected in April 2024 from Guizhou Province, Yunnan Province, and Zhejiang Province, China, respectively, and identified with the help of Professor Nian Liu, a botanist from Zhongkai University of Agricultural and Engineering. The extract was stored in the Institute of Nature Medicine and Green Chemistry (Guangdong University of Technology, Guangzhou, China) as a voucher specimen (ZLY-20240420-001, ZLY-20240420-002 and ZLY-20240420-003). RAW 264.7 (ATCC TIB-71) and HaCaT (CLS 300493) cell lines were obtained from BOSTER (Wuhan, China). Dulbecco’s modified eagle medium (DMEM), phosphate buffered saline (PBS) and Trypsin-EDTA (0.25%) were from Gibco, Grand Island, United States. Fetal Bovine Serum (FBS) was from Vivacell (Shanghai, China). MTT (98%, Reagent grade), NO detection kit, DMSO (dimethyl sulfoxide), and 4′,6-diamidino-2-phenylindole (DAPI) solution were purchased from Beyotime (Shanghai, China). The DCFH-DA probe was from Solarbio (Beijing, China). Bovine Serum Albumin (BSA), Tween 20 (20 × TBST), Triton X-100, phalloidin and NF-κB rabbit pAb were from Sigma (St. Louis, MO, United States). The mice IL-1 assay kit, mice IL-6 assay kit, mice IFN-γ assay kit, and mice TGF-β assay kit were all purchased from Shanghai Enzyme-linked Biotechnology Co., Ltd. (Shanghai, China). Goat Anti-Rabbit IgG second antibody, IL-1β antibody, TNF-α antibody, and HRP-labeled second antibody were purchased from Sigma (St. Louis, MO, United States). Chemical reagents were analytical grade and supplied by Aladdin (Shanghai, China).

### 2.2 Extraction and metabolites analysis of three tea extracts

Ethanol extraction method was used to extract substances from tea leaves. Five grams of tea leaf powder were mixed with 150 mL of 70% ethanol, stirred for 30 min, and then were refluxed at 85°C for 50 min (Tsai and Chen, 2016). The ethanol tea mixture was then vacuum dried at 40°C, and the obtained solid extract was weighed to calculate its extraction rate. Subsequently, the three tea extracts were analyzed using ultra-high-performance liquid chromatography/quadrupole Orbitrap mass spectrometry (UHPLC-Q-Orbitrap-MS) technology. The conditions of analysis were as follows:

Chromatographic separation was performed on a Thermo Scientific Ultimate 3000 UHPLC system coupled to a Q Exactive Orbitrap mass spectrometer equipped with an electrospray ionization (ESI) source. Chromatographic separation was achieved using a Hypersil Gold C18 column (100 × 2.1 mm, 1.9 μm; Thermo Scientific) maintained at 40°C, with a 2 μL injection volume. The mobile phase, consisting of 0.1% formic acid in water (solvent A) and methanol (solvent B), was delivered at a flow rate of 250 μL/min. The gradient elution program was optimized as follows: 95% A (0–5 min), linear reduction to 60% A (5–20 min), further reduction to 10% A (20–30 min), isocratic elution to 10% A (30–35 min), rapid return to 95% A (35–37 min), and column rebalancing to 95% A (37–45 min). Mass spectrometric detection was performed in both positive and negative ionization modes, with spray voltages optimized at +3.5 and −2.5 kV, respectively. The ion source parameters were carefully optimized: sheath gas flow at 40 arbitrary units (arb), auxiliary gas flow at 10 arb, and sweep gas flow at 0 arb. The capillary and auxiliary gas heater temperatures were maintained at 320°C and 350°C, respectively. The acquisition method employed automatic polarity switching between full MS (70,000 fwhm) and data-dependent MS/MS (175,000 fwhm) scans, with an isolation width of 0.4 m/z across the mass range of 50–750 m/z. System operation and data acquisition were managed through the Xcalibur software platform (Thermo Fisher Scientific). Subsequent data processing and metabolite identification were performed using Metabolite Discoverer 3.2 software (Thermo Scientific) in conjunction with the mzCloud database (http://www.mzcloud.org).

### 2.3 Establishment of animal models

The experimental animals are healthy, disease-free KM SPF grade mice, purchased from Guangdong Experimental Animal Center, aged 5 weeks, with a weight range of 34–38 g, and male sex. All mice underwent a 1 week adaptation period before the experiment and adapted in a controlled environment (22°C, 12 h light/dark cycle). The mice underwent health screening prior to the experiment to ensure that there were no obvious physiological or behavioral abnormalities and that they met the experimental requirements. If mice experience health problems during the adaptation or experimental period, such as diarrhea, weight below 34 g, excessive sleepiness, *etc.*, they should be excluded immediately. If the mice have any congenital or acquired diseases (such as skin diseases, respiratory problems, *etc.*), they will also be excluded. The mice were randomly assigned to six groups (n = 6 each): control group (propylene glycol), positive control group (DXM), model group (2% DNCB), CA group (0.5% CA), CS group (0.5% CS), and CP group (0.5% CP). During the experimental process, especially for tissue collection or any potential pain procedures, anesthesia of mice was administered by inhaling isoflurane in the induction chamber to ensure their health and minimize pain during processing. After a 1-week acclimation period in a controlled environment at 22°C with 12 h light/dark cycle, mice had their dorsal fur shaved to expose an area of approximately 4 cm × 4 cm. From days 1–4, all groups except the control group were treated with 2% DNCB on their dorsal skin. On day 6, all groups except the control group received 0.5% DNCB in acetone-propylene glycol solution. Following model establishment, mice were treated daily. The model and control groups were administered acetone-propylene glycol solution (acetone: glycol = 1:1). The positive control group received dexamethasone cream (100 mg per mouse) in the shaved area (Tsai et al., 2022). The CA, CS, and CP groups received 200 μL of their respective treatments applied to the shaved skin. On the 11th day, photographs of the dorsal skin were taken, and the appearance was documented. For further analysis, two 5 mL EP tube-sized skin tissue samples were cut and soaked in paraformaldehyde for fixation, and left to stand at room temperature. The remaining skin tissue was frozen and stored at −80°C.

### 2.4 Hematoxylin and eosin staining

Skin tissue was fixed by immersion in 4% paraformaldehyde for 48 h, followed by paraffin embedding and microtome segmentation. Subsequent to dewaxing and rehydration, tissue sections underwent hematoxylin staining for 10 min, brief differentiation in a differentiation solution for 30 s, and eosin counterstaining for 1 min. The stained sections were then subjected to dehydration, cleaning, and mounting procedures. Digital imaging was performed using a fully automated slide scanning system (Zeiss Axio Scan. Z1), followed by image analysis using Image-Pro Plus software.

### 2.5 Toluidine blue staining

Dermal specimens obtained from lesioned dorsal regions of mice were initially immobilized by immersion in 4% paraformaldehyde solution. After fixation, tissue samples underwent paraffin embedding and microtome sectioning. The histological staining protocol began with xylene-mediated deparaffinization, followed by sequential ethanol gradient dehydration. Tissue sections were then subjected to blue toluidine staining for 2–5 min, followed by rinsing in distilled water. Microscopic differentiation was achieved using 0.1% glacial acetic acid, with the process carefully monitored under optical microscopy. The differentiation reaction was terminated by thorough rinsing under running tap water. After oven drying, the sections were cleared in xylene for 10 min and permanently mounted using neutral resin. Quantitative analysis of mast cell infiltration was performed using Image Pro Plus software following digital image acquisition through an automated scanning system.

### 2.6 Immunohistochemical staining

Following xylene deparaffinization and sequential dehydration through an ascending ethanol series, tissue sections were subjected to antigen retrieval. Endogenous peroxidase activity was quenched by 10 min incubation with 3% hydrogen peroxide, followed by PBS rinses. Non-specific binding sites were blocked with normal serum prior to overnight incubation at 4°C with primary antibodies specific to TNF-α and IL-1β. Subsequent immunodetection was performed using HRP-conjugated anti-rabbit IgG secondary antibody at 37°C. Chromogenic development was achieved through incubation of DAB substrates, with nuclear counterstaining using hematoxylin. Following mounting procedures, digital images were obtained using an automated scanning system, allowing quantitative analysis of mean optical density values.

### 2.7 Cytotoxicity evaluation

Cell viability was assessed using a tetrazolium-based colorimetric assay, which relies on the reduction of water-soluble tetrazolium salts to insoluble formazan crystals by mitochondrial dehydrogenases in metabolically active cells. RAW 264.7 macrophages, previously cryopreserved in liquid nitrogen, were revived and expanded through two to three passages to ensure optimal cellular activity prior to the experiment. For the assay, cells were seeded in 96-well plates at a density of 5 × 10^3^ cells/well. The experimental well received 100 μL of complete culture medium containing tea extract at a concentration of 50 μg/mL (Novilla et al., 2023). Control wells contain either sterile medium alone (blank control) or medium with cells but without tea extract (negative control). Following 24-h incubation, 100 μL of MTT working solution was added to each well, and plates were returned to the incubator for 4 h to allow formazan crystal formation. The reaction was terminated by careful removal of the supernatant, followed by addition of 150 μL DMSO to solubilize the formazan crystals. Plates were shaken for 15 min to ensure complete dissolution before spectrophotometric measurement at 570 nm using a microplate reader. Cellular viability was calculated as the percentage of absorbance relative to the control wells.

### 2.8 NO content detection

After 24 h of culture, the supernatant of RAW 264.7 cells was discarded, and each well was replenished with complete medium containing samples of three tea extracts at a concentration of 50 μg/mL. After 2 h of incubation, LPS at a concentration of 1 μg/mL was added to induce the model, with a blank control group and an LPS model group established simultaneously. Cell supernatant was collected and centrifuged at 800 *g*, 5 min to remove debris. Subsequently, 50 μL of the supernatant was added to a 96-well plate and mixed with an equal volume of Griess reagent. The mixture was incubated at room temperature, protected from light, for 10 min. The absorbance was then measured at 540 nm using a microplate reader. Currently, a standard curve has been generated using sodium nitrite standards. The nitrite concentration in the samples was calculated using the standard curve, and the NO content in RAW 264.7 cells was analyzed using the formula:

$$\text{NO content }=\left(\text{OD of the sample well}-\text{OD of the blank well}\right)/\left(\text{OD of the control well}-\text{OD of the blank well}\right)\times 100\%$$

### 2.9 ELISA experiment

The cell culture method was identical to that described in Section 2.8. Quantitative determination of IL-1, IL-6, IFN-γ and TGF-β levels was performed following manufacturer’s protocols for the respective commercial assay kits. All experimental data were statistically analyzed using GraphPad Prism software.

### 2.10 Immunofluorescence experiment

The cell culture method was identical to that described in Section 2.8. The medium in the 24-well plate was removed. Cellular fixation was achieved by incubating cells with 1 mL of 4% paraformaldehyde at room temperature for 15 min, followed by three PBS washes. Membrane permeability was performed using 0.5% Triton X-100 for 10 min at ambient temperature with subsequent PBS rinses. Non-specific binding sites were blocked by incubating cells with 1 mL of 5% BSA in TBST (0.1% Tween-20 in Tris buffered saline) for 1 h at room temperature. Immunostaining was initiated by overnight incubation at 4°C with 200 µL of NF-κB primary antibody (1:200 dilution in 1% BSA). Following three TBST washes, cells were incubated with 200 µL of Alexa Fluor-conjugated goat anti-rabbit secondary antibody (1:800 dilution in 1% BSA) under light-protected conditions for 1 h. After thorough TBST washing, nuclei were counterstained with 200 µL of DAPI solution for 10 min, followed by final PBS rinses. Fluorescence images were captured using an inverted fluorescence microscope (Olympus IX73, Olympus, Japan) equipped with appropriate filter sets for DAPI and Alexa Fluor 488. Imaging was performed at ×20 magnification with exposure times of 200 ms for Alexa Fluor 488 and 100 ms for DAPI channels. All imaging parameters, including gain and exposure, were kept constant across all samples to ensure consistency. Quantification of integrated optical density (IOD) was conducted using Image Pro Plus software (version 6.0).

### 2.11 Scratch migration assay

The RAW 264.7 cell concentration was adjusted to 4 × 10^5^, and they were seeded into a 6-well plate for culture until adhesion growth was achieved, after which supernatant was removed. A 200 μL pipette tip was used to scratch vertically to the bottom of the well to wash away the dislodged cells. The cells were washed with PBS three times, then 1 mL of serum-free medium containing different drugs was added to each well. After 2 h of incubation, LPS was added to achieve a final concentration of 1 μg/mL, and the time of LPS addition was designated as time zero. Cell fracture images were captured at 0 and 24 h, with three field of view selected per well. The relative migration rate was calculated as (initial crack width - post-culture crack width)/initial crack width × 100%.

### 2.12 ROS detection experiment

The cell culture method was identical to that described in Section 2.8. After an additional 24 h of culture, the supernatant was discarded, and DCFH-DA was added according to the instructions provided with the kit. After 30 min, the cells were piped out, centrifuged at 800 *g*, washed, and resuspended before being counted and imaged using an automated fluorescence cell counter. Data processing and analysis were performed using GraphPad Prism statistical software.

### 2.13 Apoptosis rate detection

HaCaT cells in good growth condition at a concentration of 1 × 10^6^/mL were added to a 6-well plate and incubated for 24 h. The supernatant was aspirated, and PBS was added. The cells were then subjected to UVB modelling at 70 mJ/cm^2^. After incubation for half an hour, the cells were treated with different drugs and incubated for 24 h. According to the instructions provided by Beyotime Biotechnology, samples were prepared as follows: Control group (Control), UVB model group (UVB), high-concentration group of CA (CA-H, 50 μg/mL), low-concentration group of CA (CA-L, 25 μg/mL), high-concentration group of CS (CS-H, 50 μg/mL), low-concentration group of CS (CS-L, 25 μg/mL), high-concentration group of CP (CP-H, 50 μg/mL), and low-concentration group of CP (CP-L, 25 μg/mL). One milliliter of ice-cold 70% ethanol was added to each well, and the cells were gently pipetted to mix and fixed overnight at 4°C. The cells were centrifuged at 1,000 *g* for 5 min to pellet the cells. The supernatant was carefully removed. Approximately 1 mL of ice-cold PBS was added to resuspend the cells. The cells were centrifuged again to pellet them, and the supernatant was removed. The bottom of the centrifuge tube was gently tapped to disperse the cells appropriately, avoiding clumping. For apoptosis detection, cells were stained with Annexin V-FITC and propidium iodide (PI) at the manufacturer’s recommended ratio, gently vortexed, and incubated for 15–30 min at room temperature in the dark. Flow cytometry was then performed under light-protected conditions using appropriate fluorescence channels (Agilent NovoCyte 2060R) (Annex V-FITC: 488 nm excitation/530 nm emission; PI: 488 nm excitation/585 nm emission). Based on Annexin V and PI fluorescence, cells were classified into distinct populations: viable (Annexin V^−^/PI^−^), early apoptotic (Annexin V^+^/PI^−^), late apoptotic (Annexin V^+^/PI^+^), and necrotic/damaged (Annexin V^−^/PI^+^). Debris was excluded based on FSC-A and SSC-A profiles, and doublets were removed via FSC-H vs. FSC-A gating. Annexin V-FITC and PI signals were plotted to distinguish live cells, early apoptotic, late apoptotic, and necrotic populations, with gating thresholds set using unstained, single-stained, and fluorescence-minus-one (FMO) controls. Compensation for spectral overlap between FITC and PI was performed using single-stained compensation beads, and the matrix was applied and verified in FlowJo software 10.8.1.

### 2.14 ROS detection by flow cytometry

Following the same modeling and grouping procedures as in the cell apoptosis experiment, after 24 h of drug incubation, DCFH-DA was diluted with serum-free culture medium at a ratio of 1:1,000. The collected cells were suspended in the diluted DCFH-DA at a concentration of 1 × 10^6^/mL and incubated in a 37°C cell culture incubator for 20 min. The mixture was gently inverted every 3–5 min to ensure thorough contact between the probe and the cells. The cells were then washed three times with serum-free cell culture medium to completely remove any DCFH-DA that had not entered the cells. Cells were trypsinized, centrifuged, and resuspended in 300 μL of 1× binding buffer. The suspension was protected from light and analyzed by flow cytometry. Cell debris was excluded based on FSC-A and SSC-A profiles, and doublets were removed using FSC-H versus FSC-A plots. Live cells were gated by excluding viability dye-positive populations. ROS-positive cells were identified through DCF fluorescence in the FITC channel, with gating thresholds set using fluorescence-minus-one (FMO) controls. Compensation was performed using single-stained compensation beads, and the matrix was applied and verified within FlowJo software 10.8.1. Unstained cells and ROS-induced positive controls were included to validate gating accuracy.

### 2.15 Antibacterial experiment

The strains used were *E. coli* (ATCC25922) and *S. aureus* (ATCC6538), obtained from the Guangdong Institute of Microbiology (Guangzhou, China). The bacteria were cultured in batches in a synthetic medium supplemented with 1% tryptophan and 0.5% yeast extract (both sourced from Shanghai Aladdin Biochemical Technology Co., Ltd.). Logarithmic phase cultures of *E. coli* and *S. aureus* were diluted separately and then mixed with test samples for the preparation of scanning electron microscopy (SEM) specimens. Samples were observed under a scanning electron microscope to investigate the dose-effect relationship of the three tea extracts on the morphology of *E. coli* and *S. aureus*.

### 2.16 RNA extraction and detection from mice dorsal skin

Transcriptome sequencing and subsequent bioinformatics analysis were performed using the Illumina HiSeq4000 platform. Raw sequencing data underwent rigorous quality control processing, which included the removal of adapter-containing reads, sequences with >10% ambiguous bases (N), poly-A tails, and reads containing >50% low-quality bases (Q score ≤ 20). The analytical pipeline consisted of several alignment steps: ribosomal RNA mapping, sequence alignment, and reference genome mapping, followed by transcript assembly and quantification of gene expression levels. Quality-filtered reads were aligned using Bowtie2 and HISAT2, with transcript assembly performed using Stringtie. Differential gene expression analysis was performed using DESeq2 in R, with significantly differentially expressed genes (DEGs) identified based on false discovery rate (FDR) criteria < 0.05 and |log_2_(fold change)! > 1. Functional annotation of DEGs was performed through a comprehensive analysis of Gene Ontology (GO) enrichment, with subsequent classification of biological processes, molecular functions, and cellular metabolites. In addition, path analysis was performed by mapping DEGs to the KEGG database for functional annotation and path classification.

### 2.17 Statistical analysis

Data processing and analysis were performed using GraphPad Prism 8.0.2 software. Results were expressed as mean ± standard deviation (X ± SD). One way ANOVA was used to compare means across multiple groups, while pairwise comparisons were made with a two-tailed Student’s t-test. Statistical significance was determined at a threshold of *p < 0.05, **p < 0.01, ***p < 0.001 and ****p < 0.0001 indicating a significant difference.

## 3 Result and discussion

### 3.1 Metabolite analysis of three tea extracts

**FIGURE 1:**
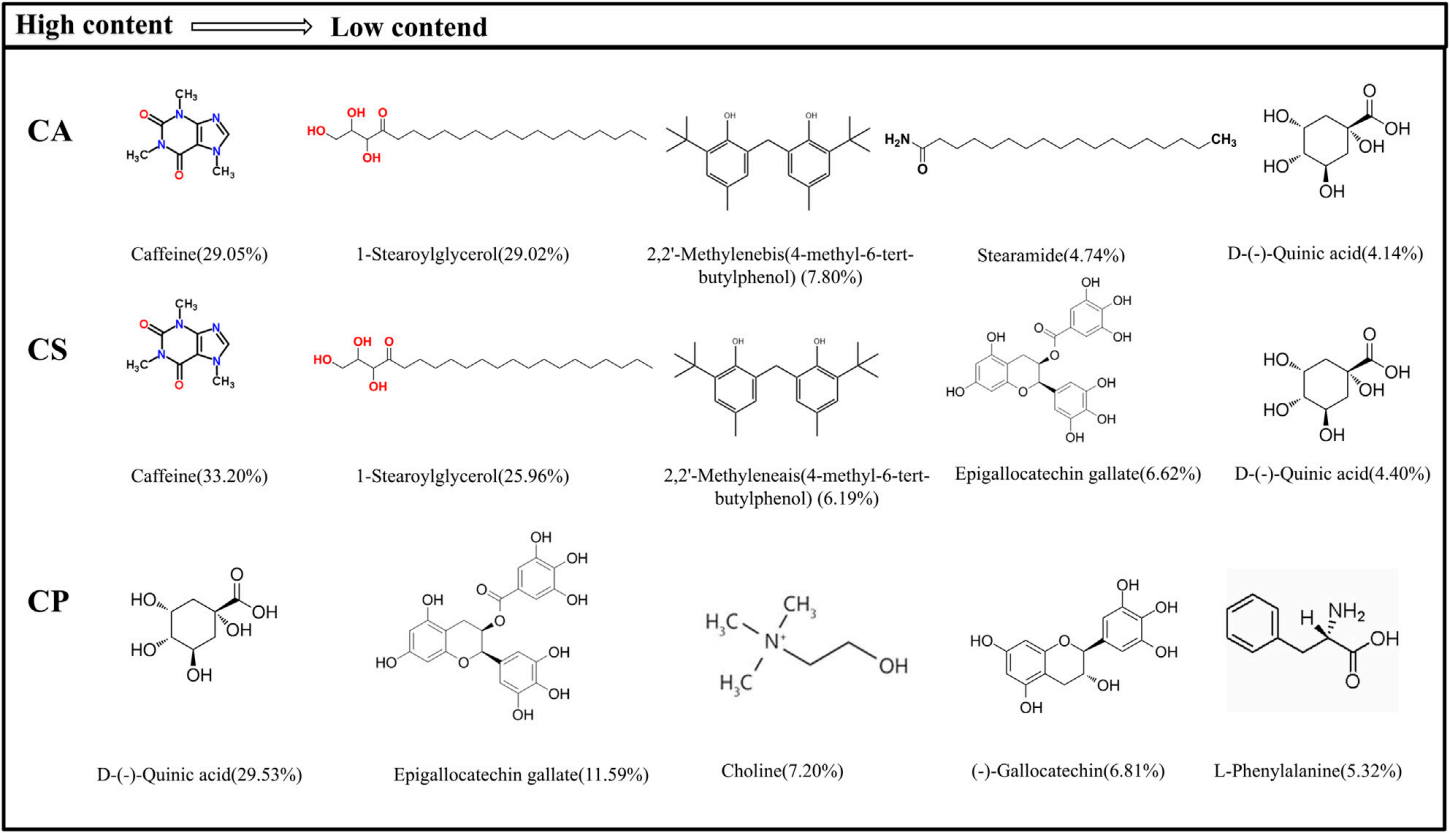
Main metabolites and their chemical structure of three types of tea extracts.

The detected metabolites are subjected to an exact molecular weight test, and their values match theoretical values. Metabolites are then searched in Metabolite Discoverer’s mz-Cloud database for further analysis and identification. Figure 1 shows the main metabolites and chemical structures of three *Camellia L.* extracts obtained from alcohol extraction, all of which are included in Supplementary Table S1 of the Supplementary Material. The data is initially processed using Thermo Xcalibur 4.1 software. Among these extracts, caffeine, 1-stearoylglycerol, 2,2′-methylenebis(4-methyl-6-tert-butylphenol), and D-()-quinic acid are common metabolites found in both CA and CS. On the other hand, CP showed higher levels of D-()-quinic acid, (−)-gallocatechin, L-norleucine, and epigallocatechin gallate than the other two varieties. According to the existing literature, key constituents such as caffeine, D-()-quinic acid, erucamide, L-norleucine, epigallocatechin gallate, choline, and (−)-gallocatechin possess anti-inflammatory properties. Moreover, CA and CS exhibit caffeine content exceeding 20%, while CP contains over 25% D-(−)-quinic acid and 10% epigallocatechin gallate. These findings highlight the potential anti-inflammatory applications of tea extracts.

### 3.2 Analysis of epidermal thickness and mast cell infiltration

**FIGURE 2:**
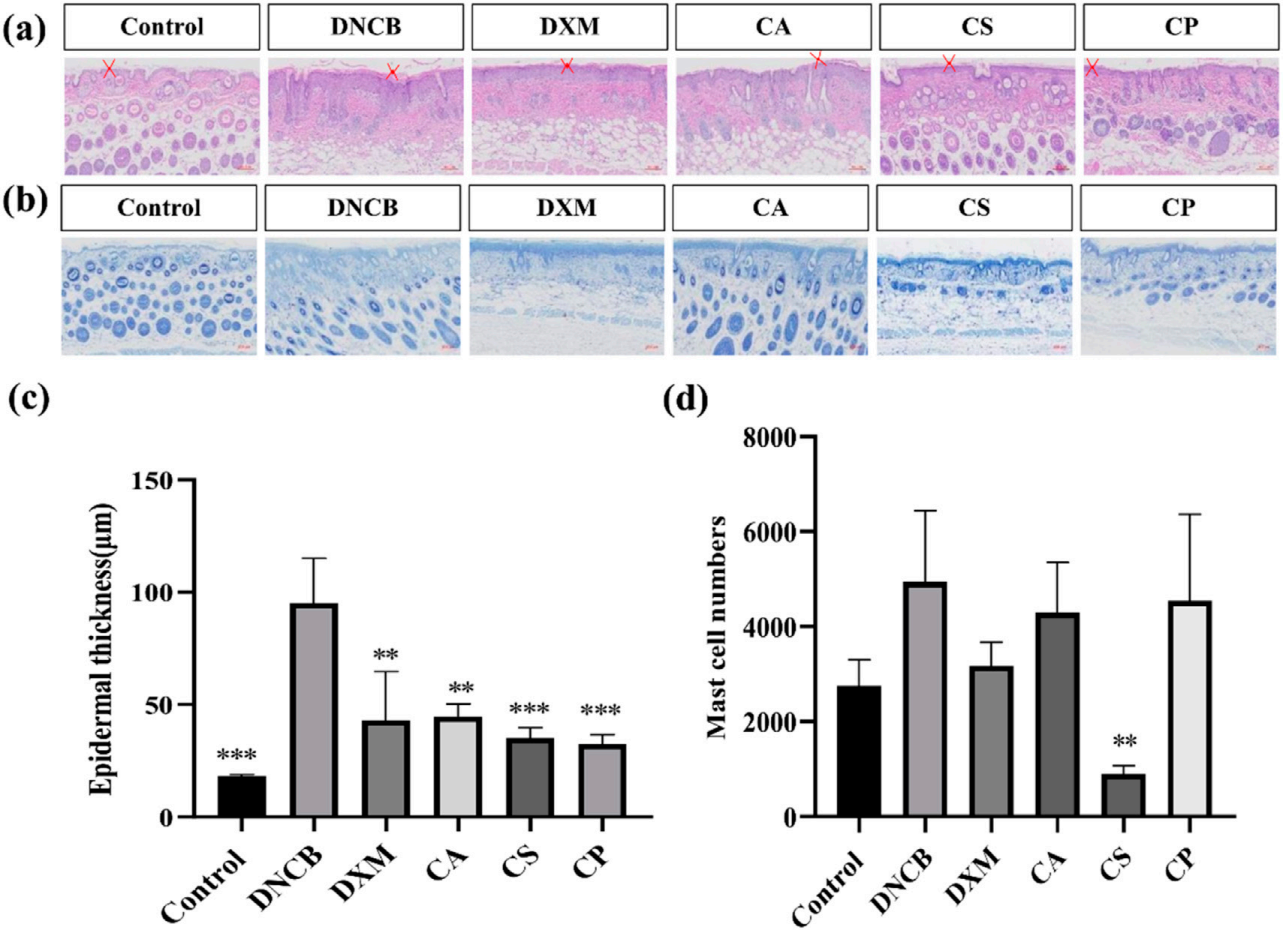
Histological analysis of HE and TB staining. **(a)** Representative images of HE staining; **(b)** Representative images of toluidine blue staining; **(c)** Quantification of epidermal thickness; **(d)** Quantification of mast cell number (n = 6, compared with model group, **p < 0.01, ***p < 0.001).

Epidermal hyperplasia, a characteristic feature of eczema-induced skin damage, indicates the efficacy of drug treatments (Guo et al., 2024; Lozano-Gerona et al., 2020). Figure 2a shows that compared to DNCB group, epidermal thickness in dexamethasone (DXM) group is reduced, and epidermal hyperplasia in CA, CS, and CP groups is also reduced, indicating that topical application of these three tea extracts can effectively inhibit DNCB-induced epidermal thickening. Furthermore, Figure 2c shows that after DNCB induction, epidermal thickness in mice increased, but after topical application of the three tea extracts, epidermal thickness is significantly reduced (*p* < 0.01), with effects comparable to those of the DXM positive control group. The results confirm that the three tea extracts can effectively reduce DNCB-induced epidermal hyperplasia.

Mast cell infiltration is a characteristic feature of skin inflammation caused by an allergic reaction, and toluidine blue staining is used to analyze the effect of CA, CS, and CP on mast cell infiltration. Figure 2b shows that following DNCB induction, there is an increase in mast cell infiltration in the skin tissue of mice. Topical applications of DXM, CA, CS, and CP all effectively reduce mast cell infiltration. Furthermore, the results in Figure 2d confirm that DXM can significantly reduce DNCB-induced mast cell infiltration into skin tissue. Of the three tea extracts, CS showed the most potent effect (*p* < 0.05), indicating that CS can effectively inhibit DNCB-induced mast cell infiltration.

### 3.3 Immunohistochemical analysis

**FIGURE 3:**
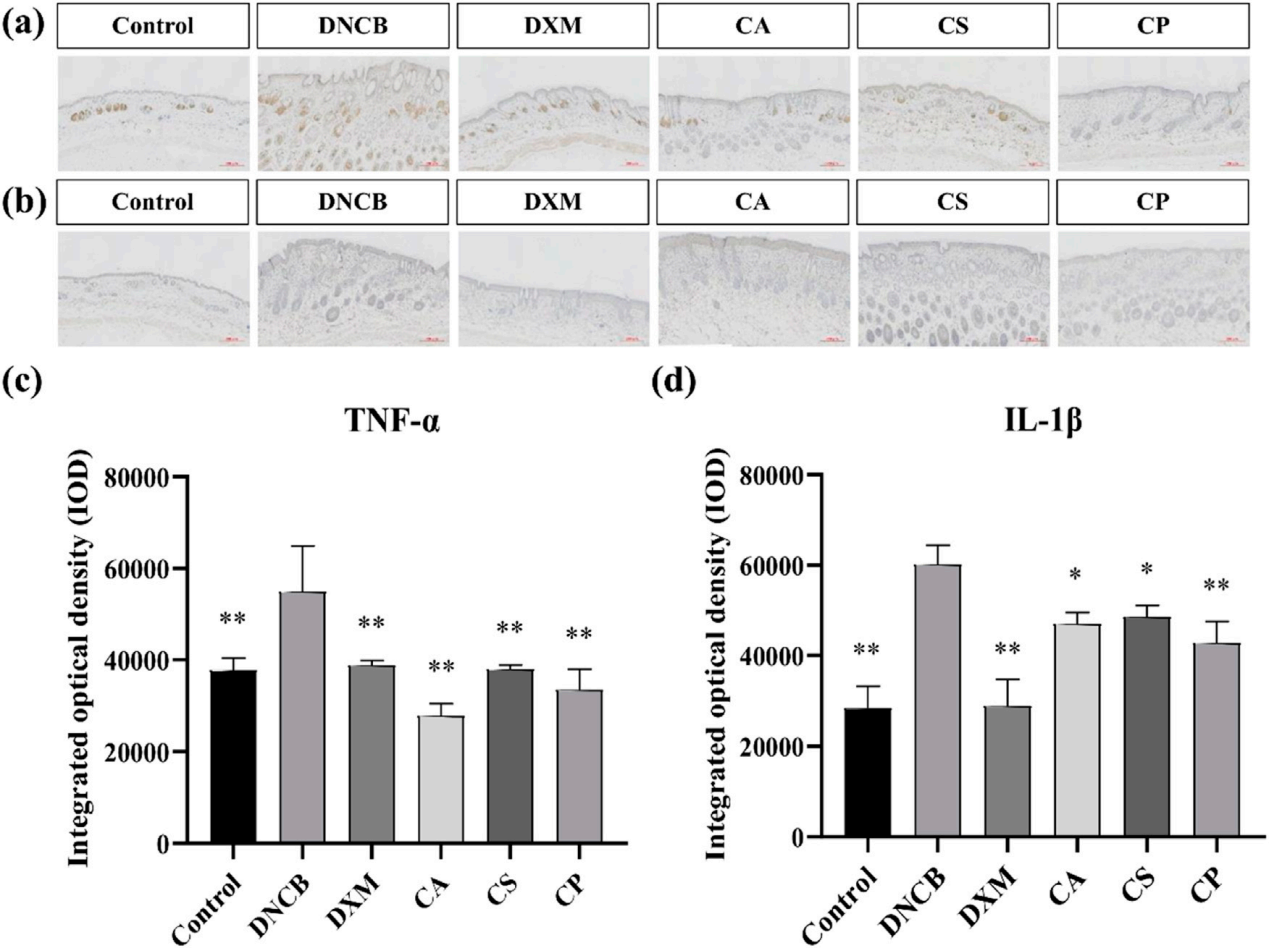
Immunohistochemical analysis of IL-1β and TNF-α expression. **(a)** Representative immunohistochemical images of TNF-α; **(b)** Representative immunohistochemical images of IL-1β; **(c)** IOD of TNF-α expression; **(d)** IOD of IL-1β expression (n = 6, compared with the model group, *p < 0.05, **p < 0.01).

IL-1β and other cytokines are essential in skin inflammation. Allergic pruritus caused by histamine triggers gene upregulation for various cytokines in the affected skin, leading to a significant increase in serum IgE levels. This, in turn, results in elevated expression of pro-inflammatory cytokines such as IL-1β and TNF-α. TNF-α, a key pro-inflammatory factor, aggravates the inflammatory response by promoting the adhesion of neutrophils and lymphocytes, causing tissue damage, vascular dilation, and increased permeability at the inflammation site (Feng et al., 2021; Song et al., 2021; Navarro-Triviño et al., 2022). Figures 3a,c show that, compared to the control group, the model group exhibits a significant increase in TNF-α expression (*p* < 0.01). In comparison to the model group, the DXM group shows reduced levels, confirming that the positive drug DXM effectively decreases TNF-α expression. Moreover, treatment with the three tea extracts also results in a reduction of TNF-α expression, with the CA group showing the most significant decrease (*p* < 0.01). Figures 3b,d reveal that, relative to the control group, the model group has a significant increase in IL-1β expression. The level of IL-1β is markedly reduced after DXM treatment (*p* < 0.01), and the expression of IL-1β also decreases following treatment with the three tea extracts, with the CP group exhibiting the best results. The findings confirm that the three tea extracts can effectively reduce the expression of skin inflammatory factors (TNF-α and IL-1β) induced by DNCB.

### 3.4 Anti-inflammatory effects of three tea extracts on LPS-induced RAW 264.7 cells

#### 3.4.1 Evaluation of cell viability and detection of NO content

**FIGURE 4:**
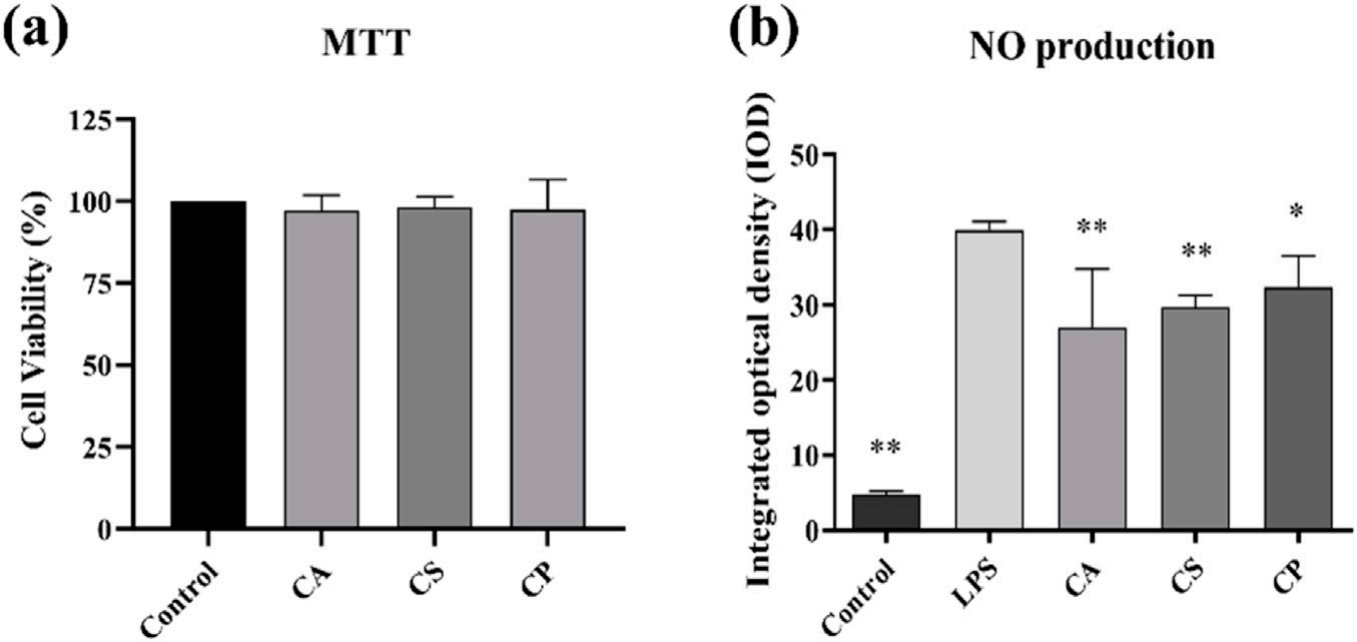
**(a)** Cell viability assessed by MTT assay; **(b)** Detection of NO content in RAW 264.7 cells (n = 6, compared with the model group, *p < 0.05, **p < 0.01).

Cell viability is assessed using a colorimetric tetrazolium-based assay. This method relies on the enzymatic activity of cellular dehydrogenases in viable cells, which catalyze the reduction of water soluble tetrazolium salts to insoluble formazan crystals. Figure 4a shows that, compared to the blank control group, the viability of RAW 264.7 cells treated with CA, CS, and CP at a concentration of 50 μg/mL is all above 90%, demonstrating that the three tea extracts at concentrations below 50 μg/mL have no significant toxicity to the cells (Novilla et al., 2023). NO is an important inflammatory factor that can cause tissue and cell toxicity, leading to oxidative damage, cell degeneration, tissue adhesion, and an increased inflammatory response. Therefore, inhibiting NO secretion by macrophages is one of the essential anti-inflammatory methods (George et al., 2019). Figure 4b shows that compared to the control group, the optical density of the model group increased significantly, confirming that LPS induces abnormal NO secretion in RAW 264.7 cells. In addition, compared to the model group, the three tea extracts significantly reduced the optical density, with CA showing the greatest reduction. These results suggest that tea extracts can effectively reduce NO secretion in RAW 264.7 cells, thus exerting an anti-inflammatory effect.

#### 3.4.2 ELISA assay for detection of inflammatory cytokine levels

**FIGURE 5:**
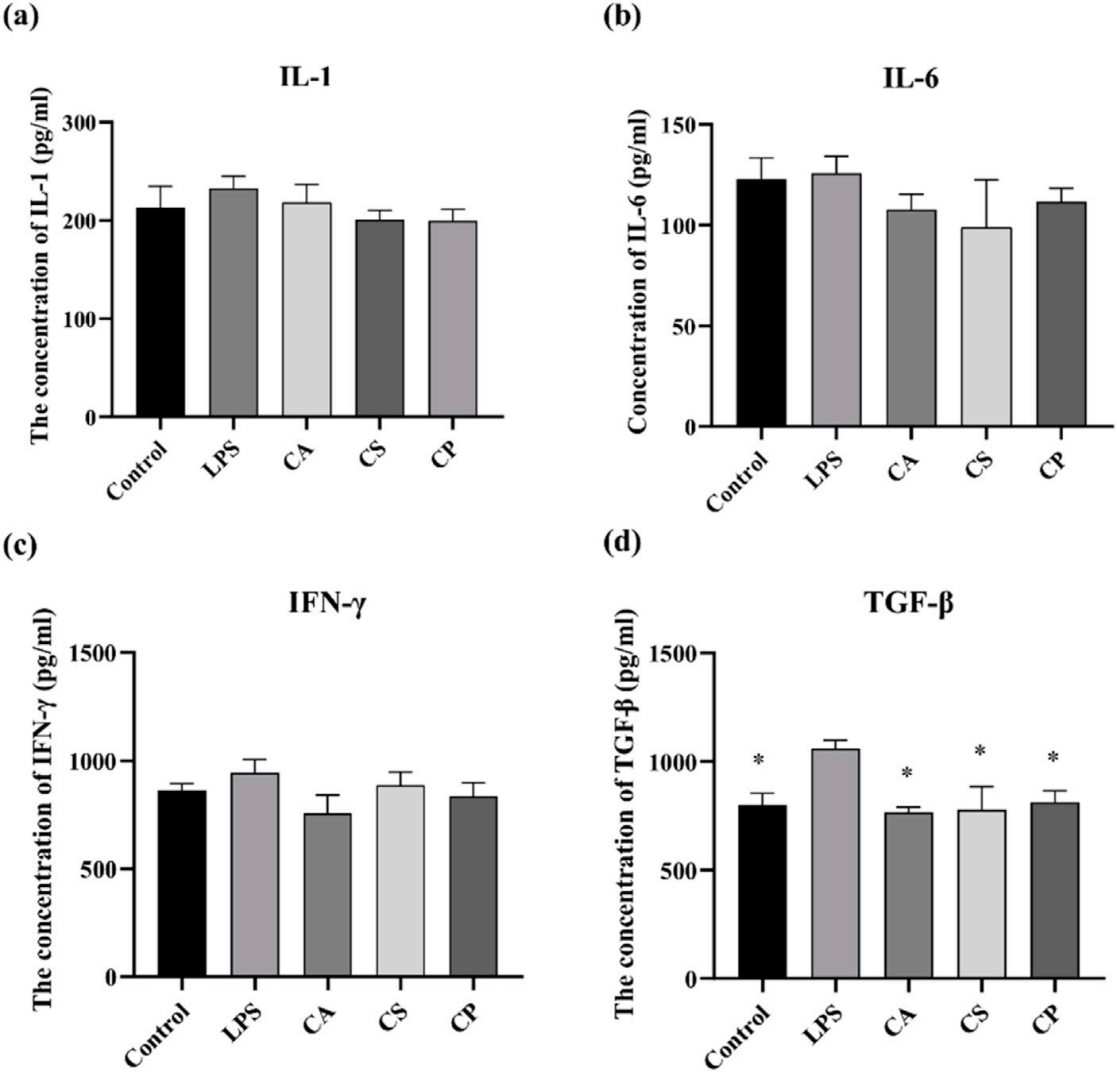
The related protein factor contents of **(a)** IL-1, **(b)** IL-6, **(c)** IFN-γ, and **(d)** TGF-β detected by ELISA (n = 6, compared with model group, *p < 0.05, **p < 0.01).

IL-1 is a pro-inflammatory cytokine that drives inflammation and is linked to autoimmune diseases, while IL-6 is a versatile cytokine involved in immune responses and is associated with chronic diseases and infections. IFN-γ is essential for antiviral and antitumor immunity, activating immune cells to combat infections, and TGF-β regulates immune tolerance, suppresses excessive inflammation, and supports tissue repair. Together, these cytokines play critical roles in balancing immune responses, maintaining tissue integrity, and regulating inflammation (Batlle and Massagué, 2019; Dinarello, 2018; Fu et al., 2023; Orange et al., 2023; Wang K. et al., 2023). The results from Figures 5a–d indicate that, compared to the blank control group, the levels of IL-1, IL-6, IFN-γ, and TGF-β in RAW 264.7 cells increase after LPS induction. Following treatment with the three tea extracts, the levels of inflammatory cytokines decrease, with a significant inhibitory effect on TGF-β (*p* < 0.05). The results suggest that the three tea extracts have the potential to modulate key pro-inflammatory factors such as IL-1, IL-6, IFN-γ, and TGF-β, indicating their anti-inflammatory properties.

#### 3.4.3 Immunofluorescence assay for observation of NF-κB levels

**FIGURE 6:**
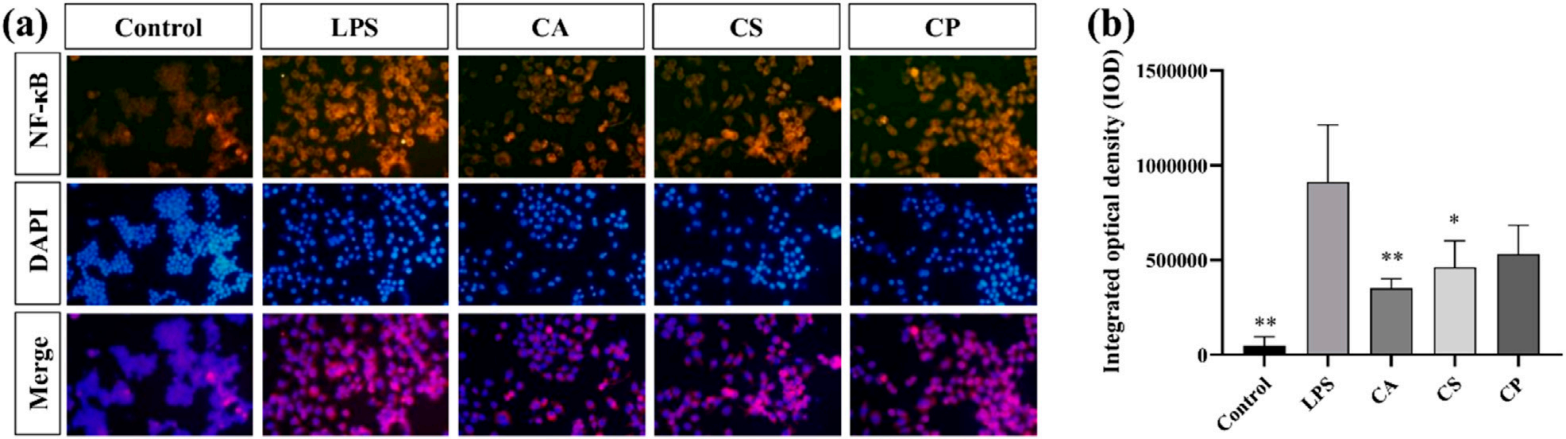
**(a)** Representative immunofluorescence images; **(b)** Quantitative analysis of fluorescence intensity of NF-κB protein (n = 6, compared with the model group, *p < 0.05, **p < 0.01).

NF-κB is crucial for cellular functions such as growth, differentiation, and apoptosis, and it is critical to inflammatory and immune responses (Dong et al., 2020; Lozano-Gerona et al., 2020; Guo et al., 2024). This signaling pathway promotes the expression and release of inflammatory factors like TNF-α and IL-6, thus contributing to inflammation. Suppressing NF-κB expression can help reduce inflammation (Li et al., 2015; Gäbelein-Wissing et al., 2015). In Figure 6a, the immunofluorescence image shows a significant increase in fluorescence intensity after LPS stimulation, indicating activation of the signaling pathway. After treatment with tea extracts (CA, CS, CP), the fluorescence intensity of NF-κB was significantly reduced, and the inhibitory effect of CA extract was the most significant (*p* < 0.01). DAPI staining showed no significant changes, ensuring that the fluorescence changes were specific to NF-κB protein. The merged images further confirmed the co localization of NF-κB and DAPI. The nuclear translocation of NF-κB was more pronounced in the LPS treatment group, while this co localization was significantly reduced after tea extract treatment, indicating that tea extract exerts anti-inflammatory effects by inhibiting the activation of NF-κB. As shown in Figure 6b, compared to the control group, LPS stimulation significantly promotes the secretion of inflammatory factors, and the expression level of NF-κB protein is also markedly increased, indicating that LPS successfully activates the inflammatory response and induces the activation of the NF-κB signaling pathway. The results demonstrate that the three tea extracts exert their anti-inflammatory effects by inhibiting the activation of the NF-κB signaling pathway, confirming their potential in anti-inflammation.

#### 3.4.4 Cell migration assay and detection of ROS levels

**FIGURE 7:**
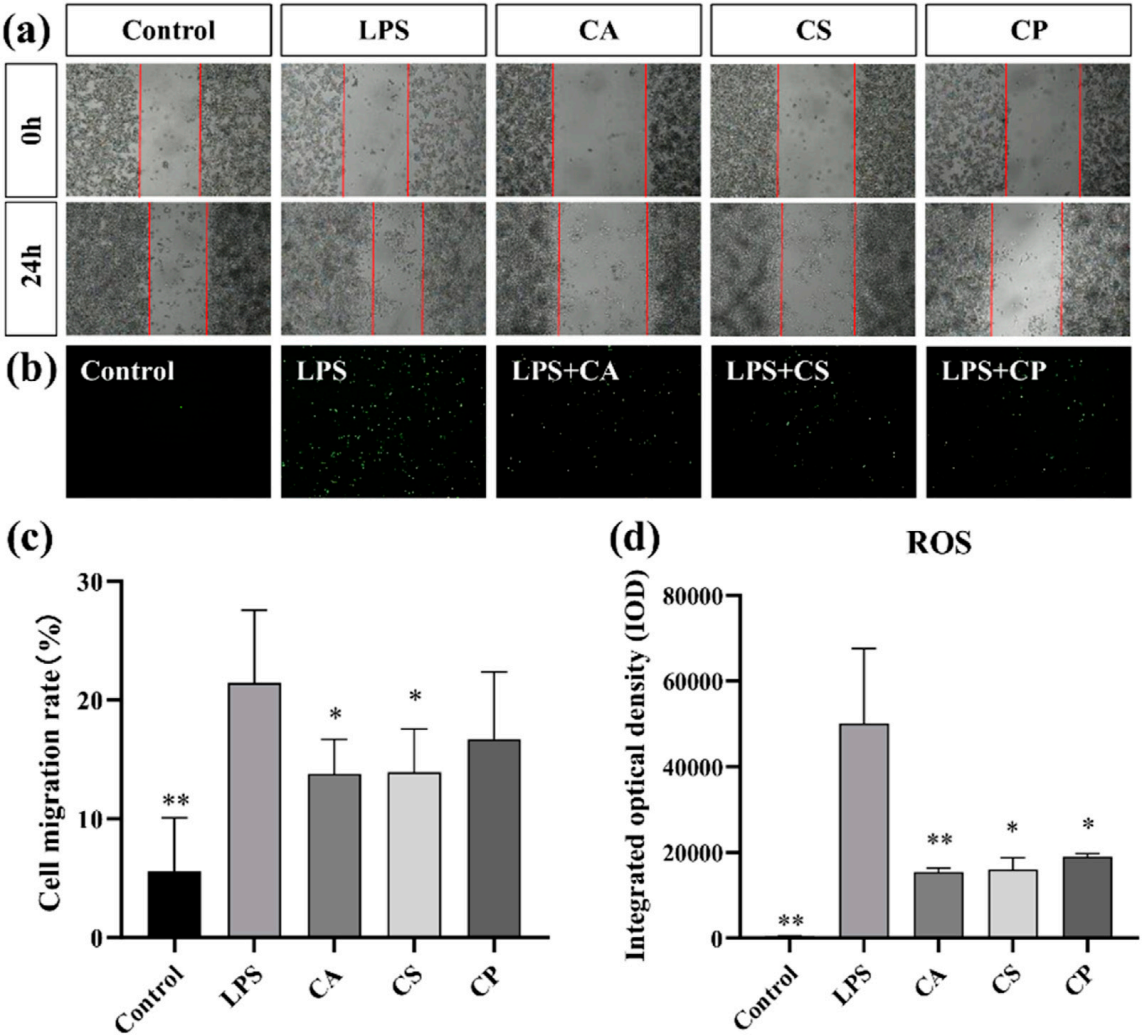
**(a)** Representative images of cell migration assay; **(b)** Representative images of intracellular ROS levels; **(c)** Quantitative analysis of cell migration rate; **(d)** Quantitative analysis of intracellular ROS levels (n = 6, compared with model group, *p < 0.05, **p < 0.01).

Scratch assay is a commonly used *in vitro* experimental method that simulates cell migration behavior during wound healing. It allows for direct observation of the speed and extent of cell migration into the buffer zone, thus assessing cell migration capacity (Martinotti and Ranzato, 2020). As shown in Figure 7a, compared to the blank control group, cell migration rates in the LPS group increased, indicating that LPS successfully induced an inflammatory response in RAW 264.7 cells, thereby promoting cell migration behavior. This result is consistent with the biological characteristics of macrophages migrating towards injury or infection sites during an inflammatory response, further confirming the effectiveness of LPS as an inflammatory stimulant. However, the results in Figure 7c show that after treatment with the three tea extracts (CA, CS, and CP), the cell migration rate decreased: CA reduced the migration rate to 13.78% (a 35.7% decrease compared to the LPS group), CS reduced it to 13.90% (35.2% reduction compared to the LPS group), and CP reduced it to 16.67% (22.2% reduction compared to the LPS group). Among them, CA and CS showed significant inhibitory effects (*p* < 0.05). This result suggests that all three tea extracts can effectively inhibit the inflammatory response induced by LPS in RAW 264.7 cells, thereby reducing their migration capacity, with CA and CS exhibiting more potent efficacy in suppressing excessive macrophage migration–a key step in mitigating inflammatory cascade amplification.

ROS, a by-product of oxygen metabolism, influences cell signaling and homeostasis. Increased ROS levels contribute to oxidative stress and inflammation (Sul and Ra, 2021; Maskey et al., 2024). As shown in Figure 7b, ROS levels in RAW 264.7 cells increased after LPS stimulation compared to the control group, indicating that LPS successfully induced an oxidative stress response in RAW 264.7 cells. Furthermore, as shown in Figure 7d, compared to the model group, intracellular ROS levels are significantly reduced after treatment with the three tea extracts (*p* < 0.05), with CA exhibiting an extremely significant antioxidant effect (*p* < 0.01). This result suggests that all three tea extracts can effectively scavenge excess ROS in RAW 264.7 cells, alleviating the oxidative stress response induced by LPS, indicating their potential application in the treatment of oxidative stress. This reduction in ROS levels could be linked to decreased migration of RAW 264.7 cells, suggesting that the antioxidant properties of tea extracts may inhibit oxidative stress response and, in turn, reduce cell migration. In conclusion, the decrease in ROS levels (Figure 7d) after treatment with tea extracts is consistent with the reduced cell migration observed in Figures 7a,c, supporting the hypothesis that inhibition of cell migration by tea extracts is partly mediated by their antioxidant effects in reducing oxidative stress.

### 3.5 Flow cytometry analysis of the effects of three tea extracts on apoptosis rate and ROS levels in HaCaT cells induced by UVB

**FIGURE 8:**
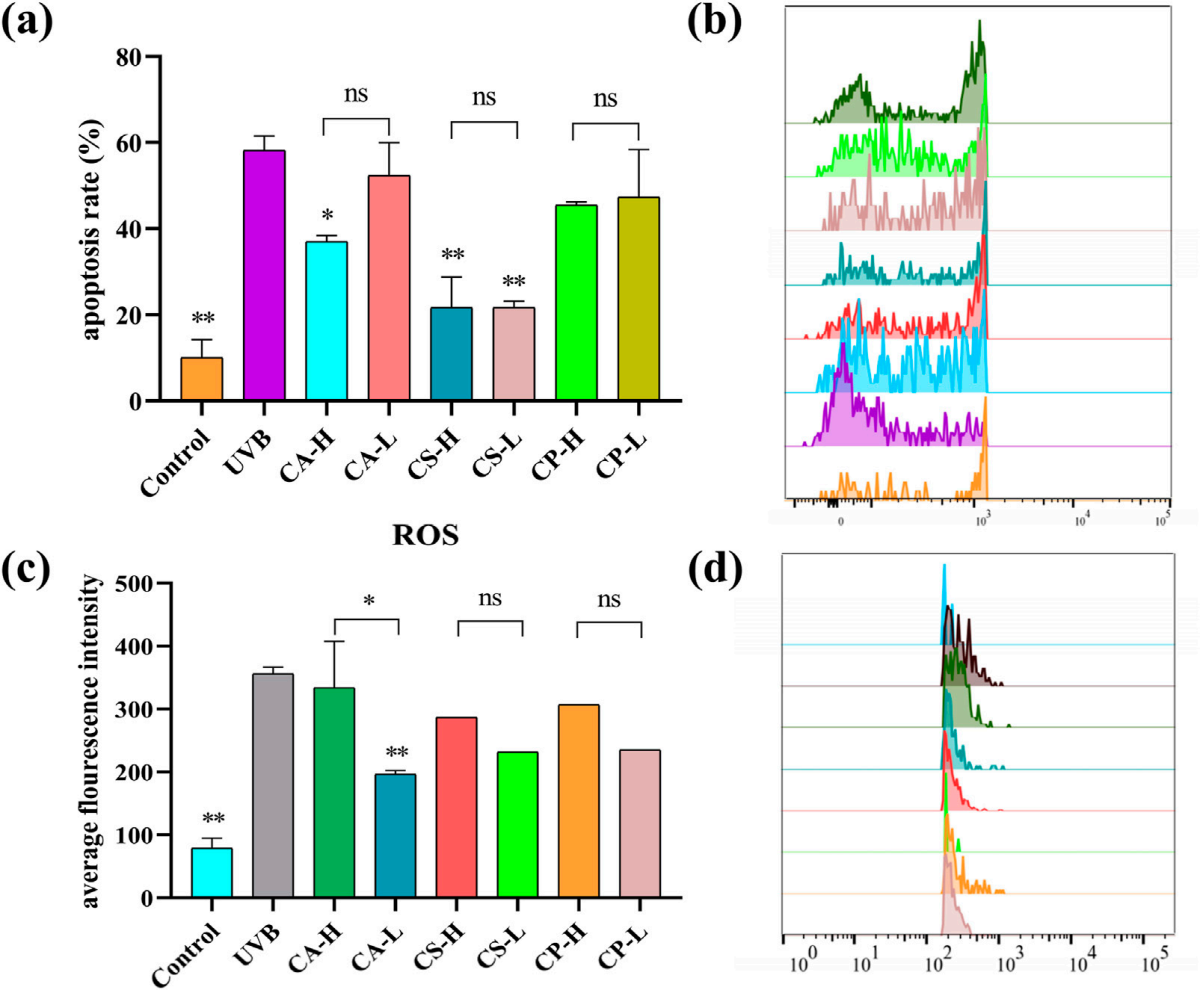
Flow cytometry analysis of apoptosis rate and ROS levels in HaCaT cells. **(a)** Histogram showing the statistical analysis of apoptosis rates; **(b)** Representative flow cytometry plots depicting the shift in apoptotic signal peaks across different groups; **(c)** Histogram illustrating the average fluorescence intensity of ROS; **(d)** Representative flow cytometry plots showing the shift in ROS fluorescence signal peaks across different groups (n = 6, compared with UVB group, *p < 0.05, **p < 0.01).

The relationship between ROS and cell apoptosis is complex and intimate. At physiological levels, ROS acts as signaling molecules involved in regulating processes such as cell proliferation and differentiation; however, when ROS is in excess, it can induce oxidative stress. This stress can damage mitochondria, the endoplasmic reticulum, and DNA, activating caspase cascades, the p53 pathway, as well as the MAPK and NF-κB signaling pathways, ultimately leading to the induction of cell apoptosis (Wang B. et al., 2023). To comprehensively evaluate the protective effects of three tea extracts (CA, CS, CP) against UVB radiation-induced photoaging damage in HaCaT cells, this study employed flow cytometry to quantify cell apoptosis rate and ROS levels. As shown in Figures 8a,b, compared to the blank control group, the UVB group showed an increased rate of cell apoptosis, while the treatment groups with the three tea extracts are able to inhibit UVB-induced cell apoptosis to varying degrees. Among them, CS showed the most superior protective effect, with its high concentration treatment group showing significantly lower cell apoptosis rate than CA and CP (*p* < 0.01). In terms of ROS detection, flow cytometry results in Figures 8c,d reveal a significant rightward shift in the fluorescence signal peak of the UVB group, indicating that UVB radiation successfully induces excessive ROS production in HaCaT cells. Notably, the fluorescence intensity of the treatment groups with the three tea extracts shows varying degrees of reduction, with the CA low concentration group showing significant ROS scavenging capacity (*p* < 0.05). However, with the exception of the CA group, the remaining treatment groups do not achieve statistical significance compared to the model group, which may be related to the prolonged interval between the loading of the DCFH-DA fluorescent probe and the samples during the experiment, leading to photobleaching in some samples. These results confirm that the three tea extracts may inhibit HaCaT cell apoptosis by scavenging abnormal ROS generation, demonstrating their protective role in photoaging.

### 3.6 Antibacterial ability of three types of tea extracts

**FIGURE 9:**
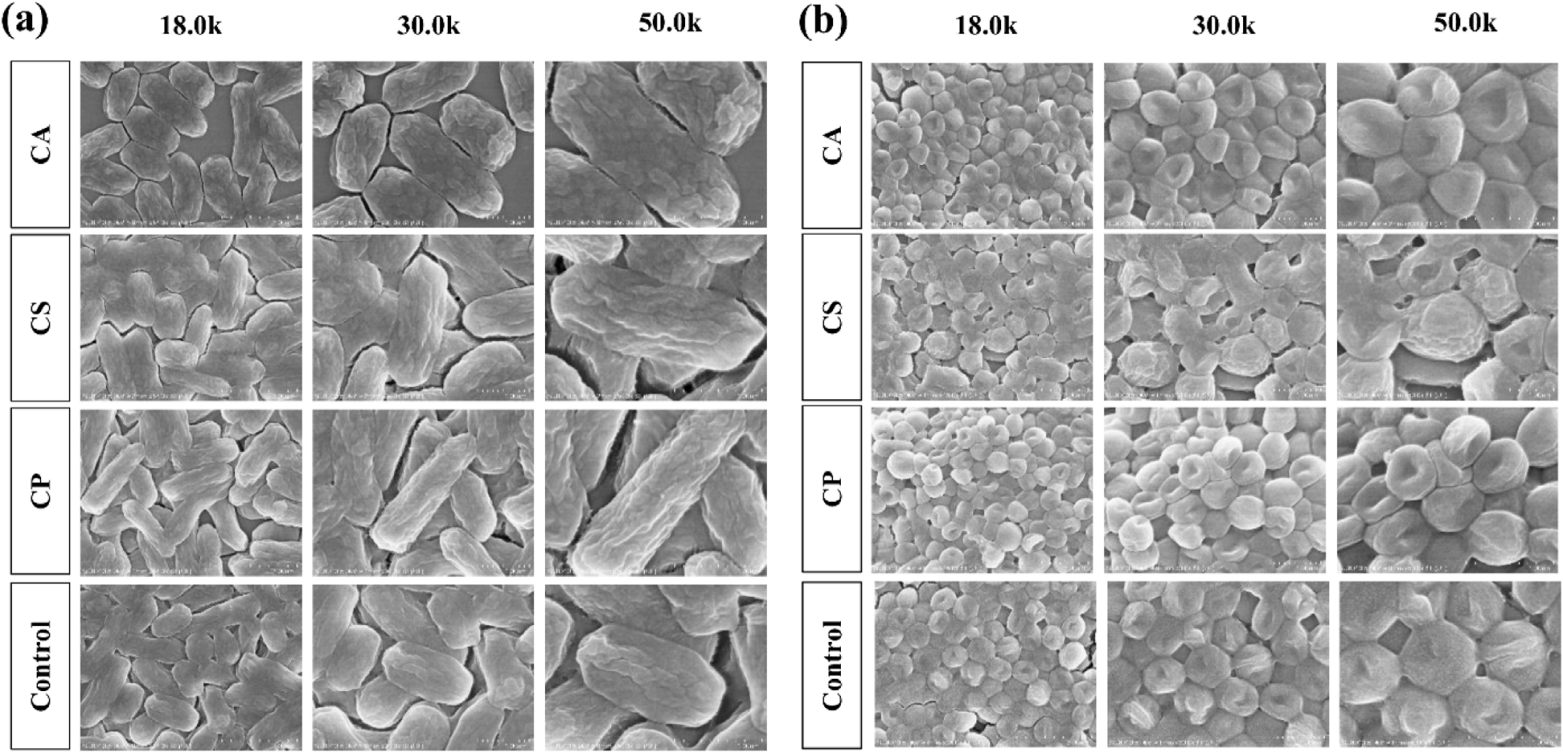
The morphological effects of three types of tea extracts on **(a)** *E. coli* and **(b)** *S. aureus.*

The bacterial cell wall undergoes dynamic changes such as expansion, remodeling, and fragmentation during growth and division, which are crucial mechanisms for bacterial adaptation to the environment and survival. As the external structure of bacteria, the cell wall not only provides physical protection, but also maintains a regular shape to support osmotic balance between the intracellular and extracellular environments, ensuring normal growth and reproduction under complex conditions (Khan et al., 2022). As shown in Figures 9a,b Under normal growth conditions, *E. coli* exhibits a typical ellipsoidal shape with an intact and regular cell wall structure, while *S. aureus* appears as a smooth, plump disc, indicating good cell wall integrity. However, compared to the blank control group, the treatment groups with the three tea extracts (CA, CS, and CP) all affect the morphology of both *E. coli* and *S. aureus*. Among them, CP has the most significant impact on the morphology of *E. coli*, transforming it from a regular ellipsoidal shape into elongated rod-like structures, while CA has the greatest effect on *S. aureus*, changing its shape from a disc to an irregular form with surface shrinkage and deformation. These morphological changes suggest that the three tea extracts may disrupt the integrity of the bacterial cell wall or interfere with its dynamic remodeling process, thus affecting bacterial division and growth. This finding provides evidence for their potential as antibacterial agents.

### 3.7 Analysis of differentially expressed genes and gene expression level

**FIGURE 10:**
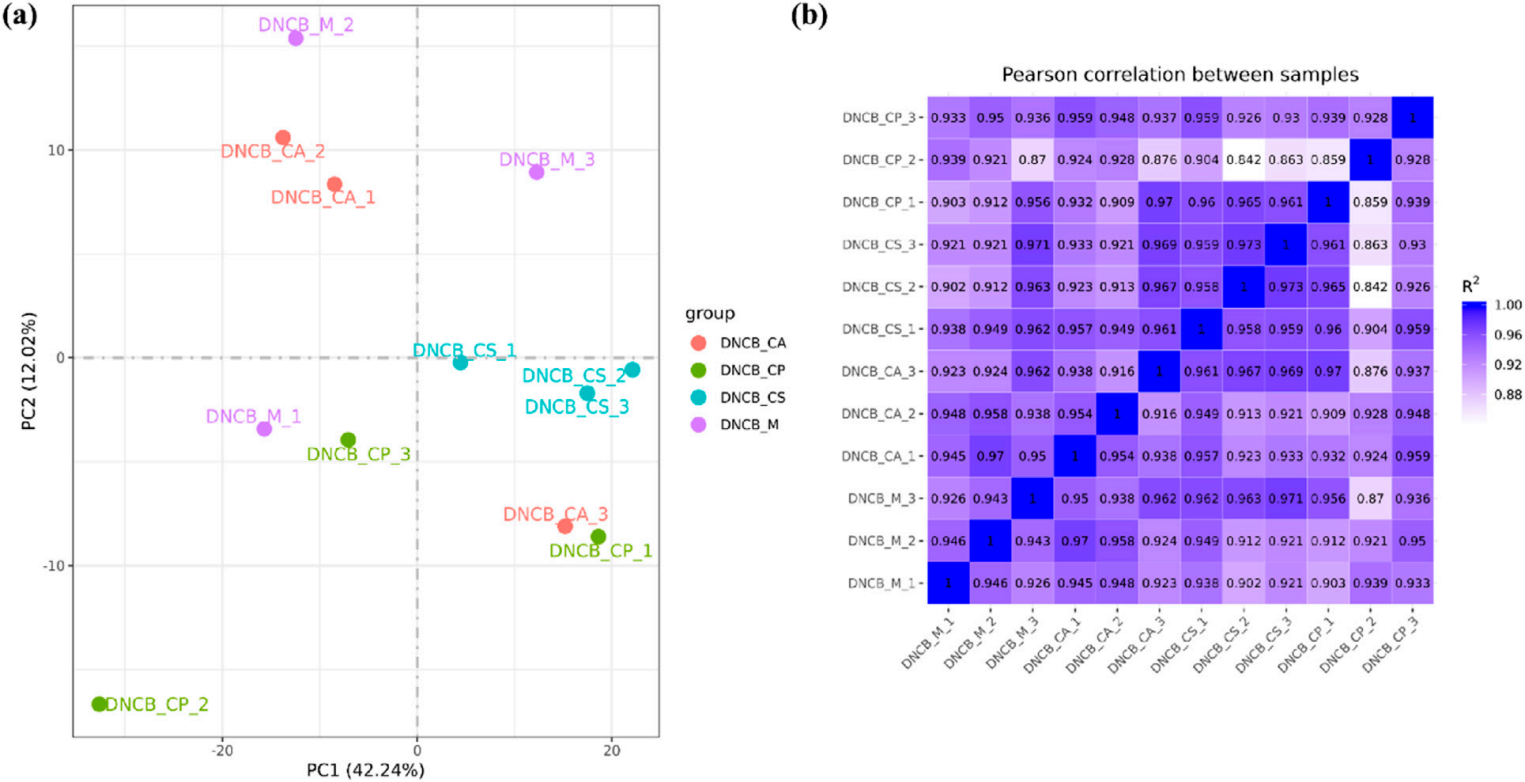
**(a)** PMA diagram; **(b)** Sample correlation.

Principal Metabolite Analysis (PMA) is used to analyze the total genetic difference of samples and the degree of similarity between samples within a group. The correlation of samples from each group is normal and there are no variation samples in Figure 10a. The correlation of gene expression levels between samples is an important index to test the reliability of the experiment and the rationality of sample selection. The closer the correlation coefficient is to 1, the higher the similarity of expression patterns between samples, and the R2 between biological repeat samples is generally required to be greater than 0.8 at least. The samples in each group have good similarities, which proves that the biological repeatability in the group is good as shown in Figure 10b.

**FIGURE 11:**
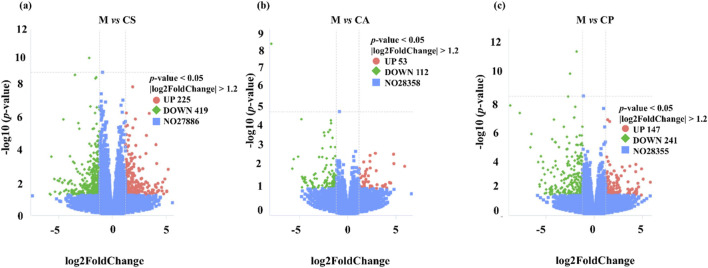
Volcanic plot of **(a)** M vs. CS, **(b)** M vs. CA, and **(c)** M vs. CP (*p* < 0.05).

We identified multiple differentially expressed genes (DEGs) through RNA extraction and transcriptome analysis, which are closely related to immune response, inflammatory response, and skin barrier function in eczema. A volcanic plot (Figure 11) was used to compare the number of upregulated and downregulated DEGs across each group, indicating significant differences in expression across all groups (*p* < 0.05). The plot also shows the number of common and unique DEGs between comparison groups (*p* < 0.05, |log_2_ FC| > 1). For example, certain DEGs may regulate Th2-type immune responses, affecting the secretion of cytokines such as IL-4, IL-5, IL-13, and thus participating in the immune mechanism of eczema. Additionally, DEGs may enhance or inhibit skin inflammatory responses by regulating the NF-κB or MAPK pathways. Damage to the skin barrier in eczema is associated with differential expression of genes such as Filaggrin and Tight Junctions, which affect the function of the skin barrier and further exacerbate the occurrence and development of eczema. Some DEGs may also be associated with overall health, immune regulation, and tissue repair processes, affecting the severity of eczema. We will further explore the biological roles of these genes in future research, especially their application in eczema, and further elucidate their functions through KEGG pathway analysis.

**FIGURE 12:**
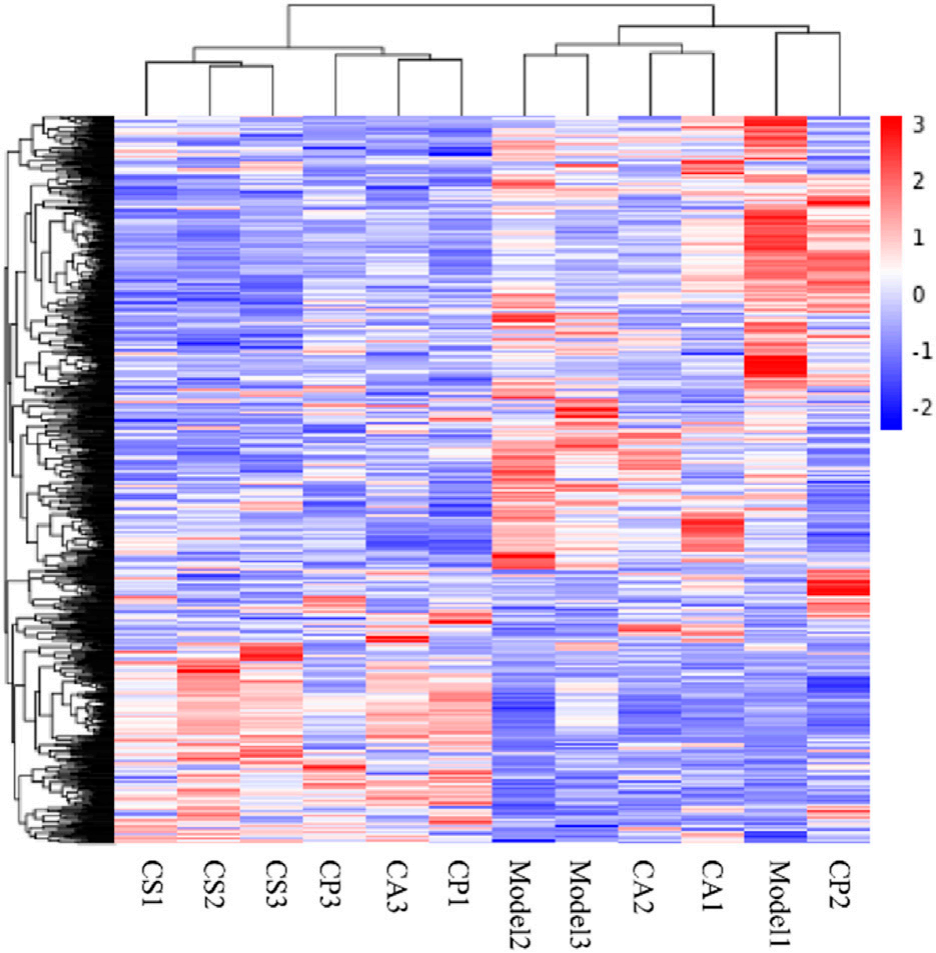
Cluster heat map. Red indicates high gene expression in the sample and blue indicates low gene expression.

After screening for skin model genes, Fragments Per Kilobase Million (FPKM) values of the genes are analyzed by mainstream hierarchical clustering. In the cluster heat map (Figure 12), each column represents a sample, and each row represents a gene. Significantly expressed genes in the model group are not significantly expressed in the treatment group, and the phenomenon is most obvious in the CS treatment group.

### 3.8 Analysis of the gene function

**FIGURE 13:**
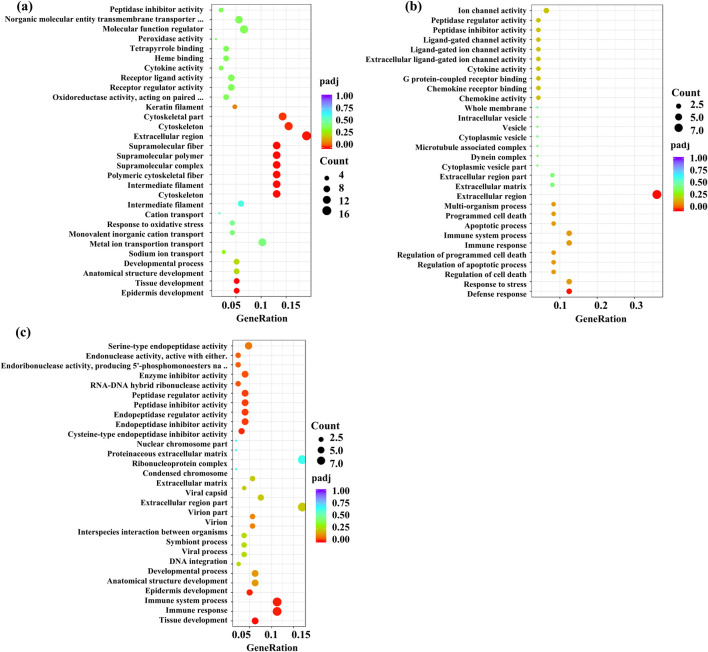
Gene Ontology (GO) functional enrichment analysis for different comparison groups: **(a)** M versus CS, **(b)** M versus CA, and **(c)** M versus CP. The bar charts represent significantly enriched GO terms categorized into biological process, molecular function, and cellular component.

GO (gene ontology) collects standard terms to define and describe the gene functions of various species. Gene products are defined according to the three related categories of biological process, cellular metabolite, and molecular function, and each category contains more specific terms. GO analysis returns a *p-*value for each GO with the presence of differential genes, and a small *p-*value indicates the enrichment of differential genes in the GO. The GO functional enrichment region includes the extracellular region and cytoskeleton in the M vs. CS group, the extracellular region in the M vs. CA group, and the same in the M vs. CP group (Figures 13a–c).

**FIGURE 14:**
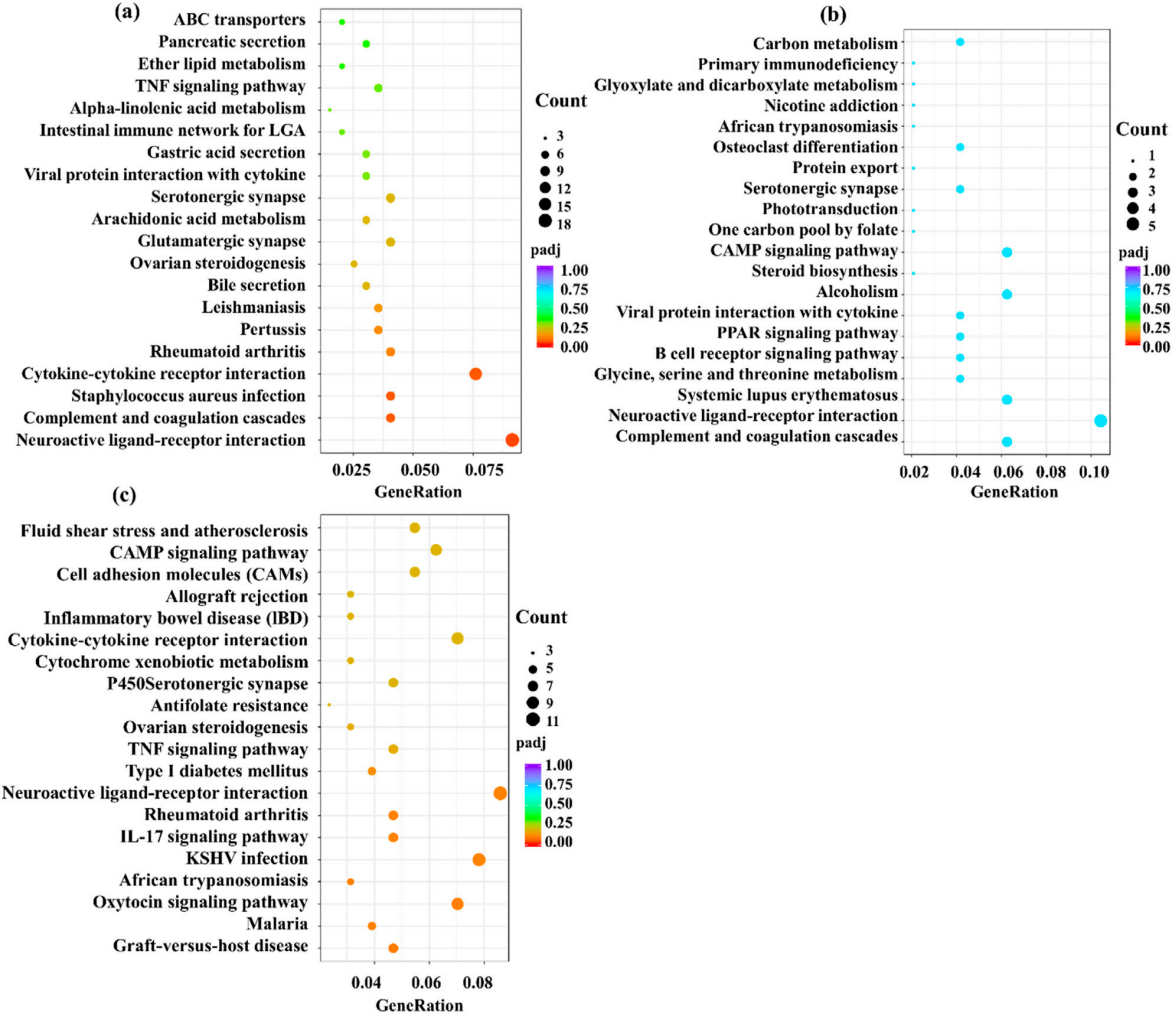
Kyoto Encyclopedia of Genes and Genomes (KEGG) pathway enrichment analysis for the comparison groups: **(a)** M versus CS, **(b)** M versus CA, and **(c)** M versus CP. The plots display significantly enriched pathways with corresponding enrichment scores and statistical significance.

KEGG (Kyoto Encyclopedia of Genes and Genomes) is a comprehensive database integrating genomic information, chemical information, and biochemical system function information. A small *p-*value indicates that differential genes are enriched in this pathway jointly. As shown in Figures 14a–c, KEGG enriches 20 signaling pathways in each comparison group, which are mainly related to inflammatory response, immune regulation, hormone synthesis and metabolism, vitamin regulation, and other signaling pathways. The active metabolites of the three essential oils may regulate multiple signaling pathways through multiple targets such as IL-1β, TNF-α, IL-4, IL-6, IFN-γ, and IL-2 to inhibit the inflammatory response of skin eczema. The results of this study provide an important basis for further research. Specifically, it is closely related to signaling pathways such as neuroactive ligand-receptor interaction in the M vs. CS group, cAMP signaling pathway and systemic lupus erythematosus in the M vs. CA group, and cytokine-cytokine receptor interaction in the M vs. CP group.

## 4 Conclusion

In conclusion, extracts from CS, CP, and CA show significant potential in alleviating eczema-like symptoms in mice, as evidenced by reduced skin thickening, inflammation (including lower TNF-α, IL-1β, and mast cell infiltration), and modulation of key pathways (NO, ROS, NF-κB). They also exhibit anti-photoaging effects and low cytotoxicity. The anti-eczema mechanisms likely involve multi-pathway regulation beyond NF-κB: potential suppression of JAK-STAT (to limit inflammatory amplification) and NLRP3 inflammasome (to reduce IL-1β maturation) (Chang et al., 2023), plus Nrf2 activation (to counter oxidative stress) (Marwick et al., 2021). These actions collectively inhibit free radical overproduction, regulate cytokines, and modulate immune cell migration, mitigating excessive immune activation. Limitations include incomplete understanding of interrelated pathways (e.g., NLRP3, JAK/STAT), undefined optimal doses, and lack of clinical validation. Future research should focus on clarifying the cross-talk pathway, establishing long-term safety, and conducting clinical trials to confirm efficacy in human eczema.

## Data Availability

The original contributions presented in the study are publicly available. This data can be found here: https://doi.org/10.6084/m9.figshare.30026125.v1.
