# Supplementary material for: Anti-eczema potential of three tea extracts: mechanisms of anti-inflammatory, antibacterial, antioxidant, and immunomodulatory effects
Source: Front Pharmacol. 2025 Sep 22;16:1595573. doi: 10.3389/fphar.2025.1595573 (PMC12498000; doi:10.3389/fphar.2025.1595573)
Supplement: Supplementary file 1 [file Supplementaryfile1.docx]

***Supplementary Material***

Table S1 UPLC-Q-TOF-MS analysis of three types of tea extracts

| NO. | Tentative identiﬁcationd | | Relative content% | | | | RT (min) |  | Formula | CAS | Mass (m/z) | Adducta | Main fragment  ions(relative intensity) |
| --- | --- | --- | --- | --- | --- | --- | --- | --- | --- | --- | --- | --- | --- |
|  |  |  | CA | CS | CP | |  |  |  |  |  |  |  |
| Ketone compounds | |  | | | | | | | | | | | |
| 1 | α,α-Trehalose | | 0.03 | 0.61 | 1.28 | | 0.94 |  | C_12_H_22_O_11_ | [99-20-7](https://www.chemsrc.com/baike/895094.html) | 342.12 | [M-H]- | 59.01375,71.01369,89.02427,179.05592 |
| 2 | (4-chlorophenyl)[4,6-dimethyl-3-(1H-pyrrol-1-yl)thieno[2,3-b]pyridin-2-yl]methanone | | 0.02 | 0.24 | 0.89 | | 1.56 |  | C_20_H_15_ClN_2_OS | [478042-93-2](https://www.chemsrc.com/baike/1523478.html) | 366.06 | [M+H]+ | 349.05 |
| 3 | Kaempferol-3-O-rutinoside | | 0.09 | 0.24 | 0.09 | | 15.66 |  | C_21_H_38_O_8_ | [17650-84-9](https://www.chemsrc.com/baike/701844.html) | 594.16 | [M+H]+ | 71.04961,85.02871,287.05405 |
| 4 | Isophorone | | 0.08 | 0.02 | 0.19 | | 16.23 |  | C_15_H_22_O_3_ | [78-59-1](https://www.chemsrc.com/baike/1032089.html) | 138.10 | [M+H]+ | 69.03403,83.04949,97.06501,139.11147 |
| 5 | Myricetin | | 0.02 | — | 0.04 | | 13.90 |  | C_15_ H_10_O_8_ | [529-44-2](https://www.chemsrc.com/baike/122124.html) | 318.04 | [M+H]+ | 153.01787,217.04898,245.04375,273.03839 |
| flavonoids compounds | | |  | | |  | | | | | | | |
| 6 | myricetin 3-O-beta-D-galactopyranoside | | 0.01 | 0.15 | 0.35 | | 13.90 |  | C_21_H_20_O_13_ | 19833-12-6 | 480.09 | [M+H]+ | 85.02874,153.01787,319.04391 |
| 7 | Morin | | 0.01 | 0.05 | 0.10 | | 14.64 |  | C_15_H_10_O_7_ | [480-16-0](https://www.chemsrc.com/baike/586469.html) | 302.04 | [M+H]+ | 137.02298,153.01784,229.04883,257.04358 |
| 8 | Rutin | | 0.04 | 0.61 | 0.19 | | 14.89 |  | C_27_H_30_O_16_ | [153-18-4](https://www.chemsrc.com/baike/894794.html) | 610.15 | [M+H]+ | 71.04961,85.0287,303.04892 |
| 9 | Astragalin | | 0.44 | 0.31 | 0.25 | | 15.65 |  | C_15_H_10_O_7_ | [480-10-4](https://www.chemsrc.com/baike/830434.html) | 448.10 | [M+H]+ | 85.02872,287.05411 |
| 10 | Kaempferol | | 0.05 | 0.01 | 0.17 | | 15.65 |  | C_25_H_33_FO_4_ | [520-18-3](https://www.chemsrc.com/baike/894792.html) | 286.05 | [M+H]+ | 153.01782,287.05408 |
| 11 | Quercetin | | 0.01 | 0.01 | 0.06 | | 17.04 |  | C_22_H_28_N_2_O_2_ | [117-39-5](https://www.chemsrc.com/baike/947030.html) | 302.04 | [M-H]- | 107.01374,121.0293,151.00346,301.03543 |
| 12 | Luteolin | | 0.17 | 0.01 | 0.04 | | 17.78 |  | C_18_H_34_O_4_ | [491-70-3](https://www.chemsrc.com/baike/950941.html) | 286.05 | [M-H]- | 285.04 |
| 13 | Grosvenorine | | — | — | 0.01 | | 15.59 |  | C_33_H_40_O_19_ | [156980-60-8](https://www.chemsrc.com/baike/1419632.html) | 740.22 | [M+H]+ | 71.04963,85.02872,287.05414 |
| 14 | Isovitexin | | 0.01 | — | 0.04 | | 14.68 |  | C_21_H_20_O_10_ | [29702-25-8](https://www.chemsrc.com/baike/165458.html) | 432.10 | [M+H]+ | 283.0592,313.06964,337.06946,367.08014 |
| 15 | Tiliroside | | — | — | 0.15 | | 2.50 |  | C_30_H_26_O_13_ | [20316-62-5](https://www.chemsrc.com/baike/313966.html) | 594.14 | [M+H]+ | 119.04909,147.04372,287.05414, |
| 16 | Vicenin II | | — | — | 0.01 | | 12.96 |  | C_27_H_30_O_15_ | [23666-13-9](https://www.chemsrc.com/baike/514697.html) | 594.16 | [M+H]+ | 295.05914,325.06952,337.0694,457.112 |
| 17 | Vicenin III | | — | — | 0.02 | | 13.71 |  | C_26_H_28_O_14_ | [59914-91-9](https://www.chemsrc.com/baike/67255.html) | 564.15 | [M+H]+ | 295.0592,325.06964,379.07999,427.10147 |
| 18 | Vitexin | | — | — | 0.01 | | 14.22 |  | C_21_H_20_O_10_ | [3681-93-4](https://www.chemsrc.com/baike/122098.html) | 432.11 | [M+H]+ | 283.05927,284.06696,313.06967,415.10123 |
| phenols | | |  | | |  | | | | | | | |
| 19 | D-(+)-Pyroglutamic Acid | | 0.98 | 0.50 | 2.40 | | 1.05 |  | C_5_H_7_NO_3_ | [4042-36-8](https://www.chemsrc.com/baike/130095.html) | 129.04 | [M+H]+ | 84.04473,84.08109,130.04965,130.08603 |
| 20 | Nicotinamide | | 0.13 | 0.14 | 0.41 | | 1.08 |  | C_6_H_6_N_2_O | [98-92-0](https://www.chemsrc.com/baike/572203.html) | 473.10 | [M+H]+ | 80.04984,123.05513 |
| 21 | Linoleoyl Ethanolamide | | 0.06 | 0.03 | 0.19 | | 23.24 |  | C_20_H_37_NO_2_ | [68171-52-8](https://www.chemsrc.com/baike/330973.html) | 323.28 | [M+H]+ | 62.0606,67.05473,81.07021,95.08569 |
| 22 | 2,2'-Methylenebis(4-methyl-6-tert-butylphenol) | | 7.80 | 6.19 | — | | 23.42 |  | C_23_H_32_O_2_ | [72672-54-9](https://www.chemsrc.com/baike/986023.html) | 340.24 | [M-H]- | 163.11 |
| 23 | 4-tert-Amylphenol | | 0.06 | 0.05 | 0.17 | | 23.42 |  | C_11_H_16_O | [80-46-6](https://www.chemsrc.com/baike/245877.html) | 164.12 | [M-H]- | 91.77166,133.63811,151.86169,159.31261 |
| polyphenol | | |  | | |  | | | | | | | |
| 24 | Neochlorogenic acid | | 0.06 | 0.35 | 0.20 | | 9.67 |  | C_16_H_18_O_9_ | [906-33-2](https://www.chemsrc.com/baike/843309.html) | 354.09 | [M-H]- | 135.045,173.0453,179.81998,191.05597 |
| 25 | Isoquercitrin | | 0.11 | 1.09 | 0.25 | | 14.88 |  | C_21_H_20_O_12_ | [21637-25-2](https://www.chemsrc.com/baike/599763.html) | 464.09 | [M+H]+ | 85.02869,303.04889 |
| 26 | Epigallocatechin gallate | | 0.17 | 6.62 | 11.59 | | 12.71 |  | C_22_H_18_O_11_ | [989-51-5](https://www.chemsrc.com/baike/663308.html) | 458.08 | [M+H]+ | 139.0387,151.03868,153.0179 |
| 27 | Pyrogallol | | 0.01 | — | 0.02 | | 1.50 |  | C_6_H_6_O_3_ | [87-66-1](https://www.chemsrc.com/baike/828534.html) | 126.03 | [M-H]- | 69.07042,72.04491,86.09676,114.09135 |
| Heterocyclic class | | |  | | |  | | | | | | | |
| 28 | Choline | | 1.47 | 0.60 | 7.20 | | 0.93 |  | C_5_H_13_NO | [62-49-7](https://www.chemsrc.com/baike/830661.html) | 103.10 | [M+H]+ | 60.08 |
| 29 | Pipecolic acid | | 0.76 | 0.12 | 0.27 | | 0.98 |  | C_6_H_11_NO_2_ | [56879-46-0](https://www.chemsrc.com/baike/685114.html) | 129.08 | [M+H]+ | 70.06561,84.08109,130.08601 |
| 30 | Adenosine | | 0.04 | 0.07 | 0.06 | | 1.26 |  | C_10_H_13_N_5_O_4_ | [58-61-7](https://www.chemsrc.com/baike/85738.html) | 267.10 | [M+H]+ | 136.06 |
| 31 | Theobromine | | 0.05 | 0.48 | 1.28 | | 3.40 |  | C_7_H_8_N_4_O_2_ | [83-67-0](https://www.chemsrc.com/baike/1026968.html) | 180.06 | [M+H]+ | 108.0557,137.08197,138.06593,181.07155 |
| 32 | Caffeine | | 29.05 | 33.20 | — | | 9.97 |  | C_8_H_10_N_4_O_2_ | [58-08-2](https://www.chemsrc.com/baike/895838.html) | 194.08 | [M+H]+ | 110.07123,138.06578 |
| 33 | Heroin-d3 | | — | 0.02 | 0.08 | | 12.39 |  | C_21_H_23_NO_5_ | [219533-68-3](https://www.chemsrc.com/baike/1571047.html) | 372.18 | [M+H]+ | 367.14 |
| 34 | Phenylethyl 2-Glucoside | | 0.01 | 0.02 | 0.03 | | 12.70 |  | C_14_H_20_O_6_ | [14861-16-6](https://www.chemsrc.com/baike/765586.html) | 284.13 | [M+H]+ | 85.02872,97.02853,127.0388,145.04916 |
| 35 | 5-(Ethylsulfonyl)-2-[(3S)-1-(4-methoxybenzyl)-3-pyrrolidinyl]-1,3-benzoxazole | | — | 0.02 | 0.14 | | 14.27 |  | C_21_H_24_N_2_O_4_S | — | 400.15 | [M+H]+ | 204.08 |
| organic acid | | |  | | |  | | | | | | | |
| 36 | L-Glutamic acid | | 1.29 | 0.84 | 2.31 | | 0.92 |  | C_5_H_9_NO_4_ | [56-86-0](https://www.chemsrc.com/baike/832391.html) | 115.03 | [M+H]+ | 56.00286,84.04474,102.0551,130.04968 |
| 37 | L-Valine | | 0.62 | 0.46 | 1.64 | | 0.97 |  | C_5_H_11_NO_2_ | [72-18-4](https://www.chemsrc.com/baike/903377.html) | 100.05 | [M+H]+ | 58.06574,59.07355,72.08122,118.08614 |
| 38 | Gluconic acid | | 0.05 | 0.07 | 0.41 | | 0.97 |  | C_6_H_12_O_7_ | [133-42-6](https://www.chemsrc.com/baike/259556.html) | 196.06 | [M-H]- | 59.01374,75.00859,87.00861,129.01921 |
| 39 | D-(-)-Quinic acid | | 4.14 | 4.40 | 29.53 | | 0.98 |  | C_7_H_12_O_6_ | [77-95-2](https://www.chemsrc.com/baike/673962.html) | 192.06 | [M-H]- | 85.02937,87.00859,93.03441,111.00861 |
| 40 | Citric acid | | 0.92 | 0.53 | 2.92 | | 1.08 |  | C_6_H_8_O_7_ | [77-92-9](https://www.chemsrc.com/baike/1027486.html) | 206.04 | [M-H]- | 85.02936,87.0086,111.0086,191.05597 |
| 41 | Succinic acid | | — | 0.02 | 0.05 | | 1.24 |  | C_4_H_6_O_4_ | [110-15-6](https://www.chemsrc.com/baike/401712.html) | 214.12 | [M-H]- | 73.02934,99.0086,117.01916, |
| 42 | L-Norleucine | | 1.55 | 0.48 | 3.00 | | 1.39 |  | C_6_H1_3_NO_2_ | [327-57-1](https://www.chemsrc.com/baike/958114.html) | 131.09 | [M+H]+ | 69.07042,72.04491,86.09676,114.09135 |
| 43 | L-Phenylalanine | | 0.71 | 0.42 | 5.32 | | 2.24 |  | C_9_H_11_NO_2_ | [63-91-2](https://www.chemsrc.com/baike/960340.html) | 165.08 | [M+H]+ | 103.05441,120.08076,131.049 |
| 44 | [3,4,5-trihydroxy-6-(3,4,5-trihydroxybenzoyl)oxyoxan-2-yl]methyl 3,4,5-trihydroxybenzoate | | — | 0.05 | 0.03 | | 9.57 |  | C_20_H_20_O_14_ | — | 484.09 | [M-H]- | 125.0242,169.01402,211.02478,271.0459 |
| 45 | N-Acetyl-DL-tryptophan | | — | 0.03 | 0.09 | | 12.85 |  | C_13_H_14_N_2_O_3_ | [87-32-1](https://www.chemsrc.com/baike/237578.html) | 246.10 | [M-H]- | 74.02461,116.03517,116.0504,203.08252 |
| 46 | 9-Oxo-10(E),12(E)-octadecadienoic acid | | 0.18 | 0.16 | 1.32 | | 22.04 |  | C_18_H_30_O_3_ | [54665-32-6](https://www.chemsrc.com/baike/1296042.html) | 294.22 | [M+H]+ | 67.05479,93.07014,179.14267,277.21555 |
| 47 | Linoleic acid | | 0.02 | 0.01 | 0.08 | | 23.92 |  | C_18_H_32_O_2_ | — | 280.24 | [M-H]- | 179.66 |
| 48 | Monoolein | | 0.58 | 0.62 | 2.37 | | 24.00 |  | C_21_H_40_O_4_ | [111-03-5](https://www.chemsrc.com/baike/830349.html) | 356.29 | [M+H]+ | 69.0704,81.07028,265.25189,339.2886 |
| 49 | 1-Stearoylglycerol | | 29.02 | 25.96 | — | | 24.43 |  | C_21_H_42_O_4_ | — | 358.31 | [M+H]+ | 57.07054,71.08604,95.08577,109.10127 |
| 50 | Palmitic acid | | 2.60 | 1.87 | 0.03 | | 24.43 |  | C_16_H_32_O_2_ | [57-10-3](https://www.chemsrc.com/baike/80973.html) | 273.27 | [M-H]- | 237.09 |
| 51 | Stearic acid | | 0.01 | 0.01 | 0.02 | | 24.86 |  | C_18_H_36_O_2_ | [57-11-4](https://www.chemsrc.com/baike/951972.html) | 284.27 | [M-H]- | 223.02 |
| 52 | Salicylic acid | | 0.06 | 0.05 | 0.15 | | 13.53 |  | C_7_H_6_O_3_ | [69-72-7](https://www.chemsrc.com/baike/414744.html) | 138.03 | [M-H]- | 295.05914,325.06952,337.0694,457.112 |
| catechins | | |  | | |  | | | | | | | |
| 53 | (-)-Gallocatechin | | 0.03 | 1.18 | 6.81 | | 7.16 |  | C_15_H_14_O_7_ | [3371-27-5](https://www.chemsrc.com/baike/765481.html) | 306.07 | [M+H]+ | 139.03857,163.03845,181.04895,195.06451 |
| cyclic amide | | |  | | |  | | | | | | | |
| 54 | NP-010776 | | 0.01 | 0.14 | 0.03 | | 14.89 |  | C_15_H_10_O_7_ | — | 302.04 | [M+H]+ | 137.02303,153.01785,229.04884,257.04364 |
| 55 | NP-020760 | | 0.01 | — | 0.04 | | 17.58 |  | C_20_H_28_O_4_ | — | 418.26 | [M+H]+ | 203.05 |
| 56 | NP-002113 | | 0.02 | 0.02 | 0.22 | | 20.75 |  | C_20_H_28_O_4_ | — | 332.20 | [M+H]+ | 280.23456,301.14050 |
| 57 | NP-008993 | | 0.04 | 0.04 | 0.09 | | 20.80 |  | C_18_H_34_O_4_ | — | 314.25 | [M-H]- | 295.23 |
| 58 | NP-021781 | | 0.04 | 0.05 | 0.17 | | 21.26 |  | C_19_H_36_O_5_ | — | 366.24 | [M+H]+ | 367.21 |
| 59 | NP-021797 | | — | 0.01 | — | | 18.96 |  | C_12_H_22_O_3_ | — | 214.16 | [M+H]+ | 237.15 |
| 60 | Stearamide | | 4.74 | 4.07 | — | | 24.17 |  | C_18_H_37_NO | [124-26-5](https://www.chemsrc.com/baike/248366.html) | 283.29 | [M+H]+ | 284.29 |
| 61 | Docosanamide | | 0.24 | 0.13 | 0.36 | | 25.68 |  | C_22_H_45_NO | [3061-75-4](https://www.chemsrc.com/baike/751292.html) | 339.35 | [M+H]+ | 57.07051,72.04485,102.09144,116.10694 |
| 62 | Erucamide | | 3.89 | 0.41 | — | | 25.13 |  | C_22_H_43_NO | [3061-72-1](https://www.chemsrc.com/baike/56682.html) | 337.33 | [M+H]+ | 81.07026,69.07039,83.08589,97.10138 |
| Other lactam | | |  | | |  | | | | | | | |
| 63 | δ-Valerolactam | | 0.01 | — | — | | 2.49 |  | C_5_H_9_NO | [675-20-7](https://www.chemsrc.com/baike/414979.html) | 99.07 | [M+H]+ | 56.05013,82.06552,100.07584 |
| carboxylate | | |  | | |  | | | | | | | |
| 64 | Methyl (2R,4S,6S,12bR)-4-(4-fluorophenyl)-2-{[2-(4-morpholinyl)ethyl]amino}-1,2,3,4,6,7,12,12b-octahydroindolo[2,3-a]quinolizine-6-carboxylate | | 0.05 | 0.07 | 0.09 | | 23.02 |  | C_29_H_35_FN_4_O_3_ | — | 506.26 | [M+H]+ | 507.27 |
| 65 | D-(-)-Quinic acid | | 4.14 | 4.40 | 29.53 | | 0.98 |  | C_7_H_12_O_6_ | [77-95-2](https://www.chemsrc.com/baike/673962.html) | 192.06 | [M-H]- | 85.02937,87.00859,93.03441,111.00861 |
| esters | | |  | | |  | | | | | | | |
| 66 | Esculetin | | 0.07 | 0.01 | 0.07 | | 11.18 |  | C_9_H_6_O_4_ | [305-01-1](https://www.chemsrc.com/baike/313977.html) | 178.03 | [M+H]+ | 123.04393,135.04378 |
| 67 | Monoolein | | 0.58 | 0.62 | 2.37 | | 24.00 |  | C_21_H_40_O_4_ | [111-03-5](https://www.chemsrc.com/baike/830349.html) | 356.29 | [M+H]+ | 69.0704,81.07028,265.25189,339.2886 |
| terpene | | |  | | |  | | | | | | | |
| 68 | Oleanolic acid | | 0.07 | — | 0.01 | | 23.63 |  | C_30_H_48_O_3_ | [28283-45-6](https://www.chemsrc.com/baike/22584.html) | 456.36 | [M-H]- | 45531732.00 |
| furan | | |  | | |  | | | | | | | |
| 69 | TOFA | | 1.30 | 1.04 | 0.26 | | 23.06 |  | C_19_H_32_O_4_ | [54857-86-2](https://www.chemsrc.com/baike/80325.html) | 324.23 | [M+H]+ | 325.23 |
| sugar alcohol | | |  | | |  | | | | | | | |
| 70 | Bis(4-ethylbenzylidene)sorbitol | | 3.13 | 2.58 | — | | 20.10 |  | C_19_H_36_O_5_ | [1003015-78-8](https://www.chemsrc.com/baike/1440044.html) | 414.20 | [M+H]+ | 119.09 |
| aldehydes | | |  | | |  | | | | | | | |
| 71 | 5-Hydroxymethyl-2-furaldehyde | | 0.02 | 0.03 | 0.15 | | 0.98 |  | C_6_H_6_O_3_ | 67-47-0 | 126.03 | [M+H]+ | 81.03387,109.02845,113.01891,127.03877 |
| 72 | 4-Hydroxybenzaldehyde | | 0.01 | 0.29 | 0.72 | | 14.74 |  | C_7_H_6_O_2_ | 123-08-0 | 122.04 | [M+H]+ | 123.04 |
| rests | | |  | | |  | | | | | | | |
| 73 | NP-000587 | | 0.08 | 0.03 | 0.06 | | 6.93 |  | C_16_H_18_O_8_ | — | 338.10 | [M-H]- | 119.05,163.03986,191.05592 |
| 74 | PEG n8 | | 0.10 | 0.13 | 0.37 | | 12.83 |  | C_16_H_34_O_9_ | — | 370.22 | [M+H]+ | 89.06001,133.08571,177.11154 |
| 75 | PEG n11 | | 0.07 | 0.05 | 0.17 | | 14.43 |  | C_22_H_46_O_12_ | — | 502.30 | [M+H]+ | 89.05999,133.08569,177.11162,503.30521 |
| 76 | PEG n12 | | 0.04 | 0.04 | 0.11 | | 14.77 |  | C_24_H_50_O_13_ | — | 546.32 | [M+H]+ | 87.04433,89.06007,133.08583,177.11179 |
| 77 | AL 8810 Methyl ester | | — | — | 0.01 | | 17.22 |  | C_24_H_30_O_6_ | [1176541-11-9](https://www.chemsrc.com/baike/1423878.html) | 438.22 | [M+H]+ | 261.1817,439.22925 |
| 78 | 6-hydroxy-3,5a,9-trimethyl-2H,3H,3aH,4H,5H,5aH,6H,7H,9aH,9bH-naphtho[1,2-b]furan-2-one | | 0.01 | — | 0.02 | | 19.23 |  | C_20_H_30_O_4_ | — | 250.15 | [M+H]+ | 251.16 |
| 79 | Methoxyacetyl fentanyl | | 0.01 | 0.01 | 0.11 | | 19.41 |  | C_18_H_32_O_4_ | — | 352.22 | [M+H]+ | 168.08 |
| 80 | (±)9-HpODE | | — | — | 0.10 | | 21.12 |  | C_18_H_32_O_4_ | [5502-91-0](https://www.chemsrc.com/baike/842912.html) | 312.23 | [M-H]- | 171.10257,211.13394,293.21237,311.17001 |
| 81 | (±)9(10)-DiHOME | | 0.34 | 0.32 | 0.79 | | 21.35 |  | C_18_H_34_O_4_ | [1215168-54-9](https://www.chemsrc.com/baike/1696670.html) | 314.25 | [M-H]- | 171.10243,185.11792,277.21719,295.22739 |
| 82 | Monolaurin | | 0.05 | 0.05 | 0.14 | | 22.11 |  | C_15_H_30_O_4_ | [27215-38-9](https://www.chemsrc.com/baike/1194335.html) | 274.21 | [M+H]+ | 297.20 |
| 83 | 5-OxoETE | | 0.13 | 0.13 | 1.00 | | 22.34 |  | C_20_H_30_O_3_ | [106154-18-1](https://www.chemsrc.com/baike/1195157.html) | 318.22 | [M+H]+ | 301.21 |
| 84 | 2,3-dihydroxypropyl 12-methyltridecanoate | | 0.05 | 0.05 | 5.13 | | 23.05 |  | C_17_H_34_O_4_ | — | 284.23 | [M+H]+ | 57.07052,71.08601,95.08573,285.24155 |
| 85 | 1,2-Dipalmitoylphosphatidylglycerol | | 0.47 | 0.35 | 1.21 | | 24.20 |  | C_38_H_75_O_10_P | [4537-77-3](https://www.chemsrc.com/baike/634045.html) | 722.52 | [M+H]+ | 745.51 |
| 86 | Oleoyl ethylamide | | 0.81 | 0.46 | 2.11 | | 24.39 |  | C_20_H_39_NO | — | 309.30 | [M+H]+ | 72.04486,114.09129 |
| 87 | Elaidic acid | | 0.12 | 0.10 | 0.41 | | 24.40 |  | C_18_H_34_O_2_ | [112-79-8](https://www.chemsrc.com/baike/445535.html) | 282.26 | [M-H]- | 273.02 |
| 88 | Tridemorph | | 0.28 | 0.19 | 0.63 | | 24.45 |  | C_19_H_39_NO | [81412-43-3](https://www.chemsrc.com/baike/1118831.html) | 297.30 | [M+H]+ | 57.07051,58.02936,102.09145 |
| 89 | {(1R,2R)-2-[(2Z)-5-(Hexopyranosyloxy)-2-penten-1-yl]-3-oxocyclopentyl}acetic acid | | — | — | 0.05 | | 12.24 |  | C_18_H_28_O_9_ | [120399-24-8](https://www.chemsrc.com/baike/915036.html) | 388.17 | [M-H]- | 203.0529,204.07527 |
| 90 | 6,8-dihydroxy-3-(10-hydroxyundecyl)-3,4-dihydro-1H-2-benzopyran-1-one | | 0.01 | — | — | | 18.83 |  | C_20_H_30_O_5_ | — | 350.21 | [M+H]+ | 351.18 |
| 91 | (3beta,9xi)-3-(beta-D-Glucopyranosyloxy)-14-hydroxycard-20(22)-enolide | | 0.01 | — | — | | 22.72 |  | C_29_H_44_O_9_ | — | 536.30 | [M+H]+ | 520.62 |
| 92 | Methanesulfonic acid | | 0.01 | — | 0.03 | | 0.98 |  | CH_4_O_3_S | [17696-73-0](https://www.chemsrc.com/baike/88198.html) | 95.99 | [M-H]- | 79.9572,94.98067 |
| 93 | EQH | | 0.01 | — | 0.07 | | 13.04 |  | C_16_H_24_N_6_O_7_ | — | 412.17 | [M+H]+ | 413.18 |
| 94 | (3R,4R)-3-({4-[(3,5-Dimethyl-1,2-oxazol-4-yl)methoxy]benzoyl}amino)-4-hydroxy-N-[4-(trifluoromethoxy)phenyl]-1-azepanecarboxamide | | — | — | 0.12 | | 13.75 |  | C_2_H_29_F_3_N_4_O_6_ | — | 562.20 | [M+H]+ | 203.05209,204.07527 |
| 95 | 1-(3,4-dichlorophenoxy)-3-({2-[(5-propylpyrimidin-2-yl)amino]ethyl}amino)propan-2-ol | | 0.02 | — | 0.46 | | 13.95 |  | C_18_H_24_C_l2_N_4_O_2_ | — | 398.13 | [M+H]+ | 202.06 |
| 96 | Gallic acid | | 0.03 | — | 0.02 | | 14.77 |  | C_7_H_6_O_5_ | [149-91-7](https://www.chemsrc.com/baike/164357.html) | 170.02 | [M-H]- | 125.02422,169.01392 |
| 97 | Caffeic acid | | 0.05 | — | — | | 12.44 |  | C_9_H_8_O_4_ | [331-39-5](https://www.chemsrc.com/baike/1099396.html) | 180.04 | [M-H]- | 90.92448,121.02946,135.04504 |
| 98 | (2R,3R,4S,5S,6R)-2-[(3Z)-hex-3-en-1-yloxy]-6-(hydroxymethyl)oxane-3,4,5-triol | | — | — | 0.02 | | 13.61 |  | C_12_H_22_O_6_ | — | 262.14 | [M+H]+ | 88.08 |

Table S2 Summary of the efficacy of each extract

| **Activation index** | **Detection model** | **CA**  **group** | **CS**  **group** | **CP**  **group** | **DXM control group** | **Optimal group** |
| --- | --- | --- | --- | --- | --- | --- |
| epidermal thickness | DNCB induced mice model | **↓↓**  **(*p*<0.01)** | ↓↓  (*p*<0.01) | ↓↓  (*p*<0.01) | ↓↓↓  (*p*<0.01) | CA/CS/CP |
| mast cell infiltration | DNCB induced mice model | ↓↓  (*p*<0.01) | **↓↓↓**  **(*p*<0.05)** | ↓↓  (*p*<0.01) | ↓↓↓  (*p*<0.01) | CS |
| TNF-α | immunohistochemistry | **↓↓↓**  **(*p*<0.01)** | ↓↓  (*p*<0.01) | ↓↓  (*p*<0.01) | ↓↓↓  (*p*<0.01) | CA |
| IL-1β | immunohistochemistry | ↓↓  (*p*<0.01) | ↓↓  (*p*<0.01) | **↓↓↓**  **(*p*<0.01)** | ↓↓↓  (*p*<0.01) | CP |
| NO | RAW 264.7 cells | **↓↓↓**  **(*p*<0.01)** | ↓↓  (*p*<0.01) | ↓↓  (*p*<0.01) | ↓↓↓  (*p*<0.01) | CA |
| NF-κB | immunofluorescence | **↓↓↓ (*p*<0.01)** | ↓↓  (*p*<0.01) | ↓↓ (*p*<0.01) | ↓↓↓  (*p*<0.01) | CA |

'↓' stands for inhibitory effect, and the more there is, the stronger the effect. The bolded annotation group represents the extract with the best effect under this indicator.


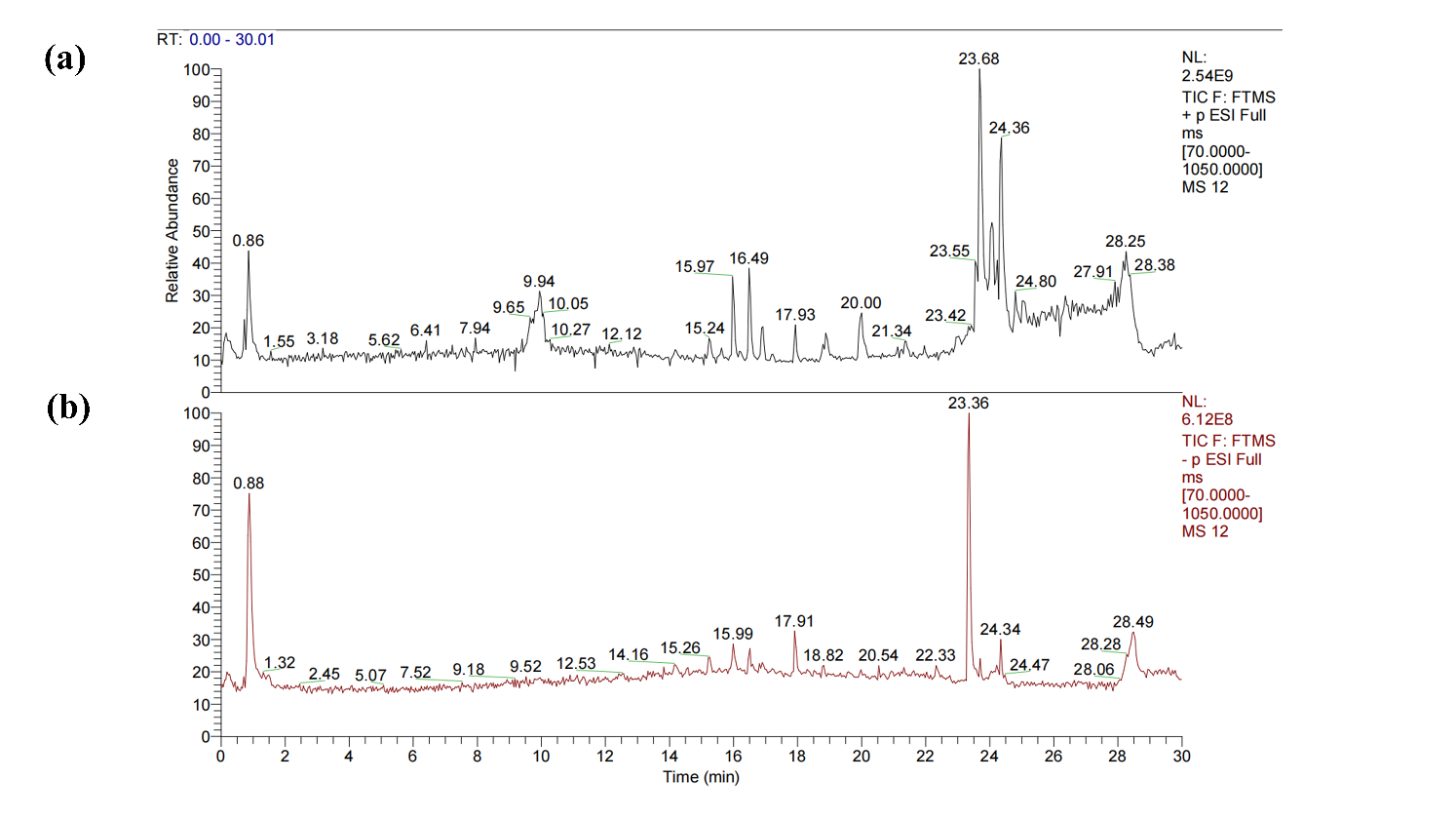


Figure S1 (a) Total ion chromatography of *Camellia arborescens Hung T. Chang, F. L. Yu & P. S. Wang (Theaceae)* (CA) extract in positive mode, (b) Total ion chromatography of CA extract in negative mode


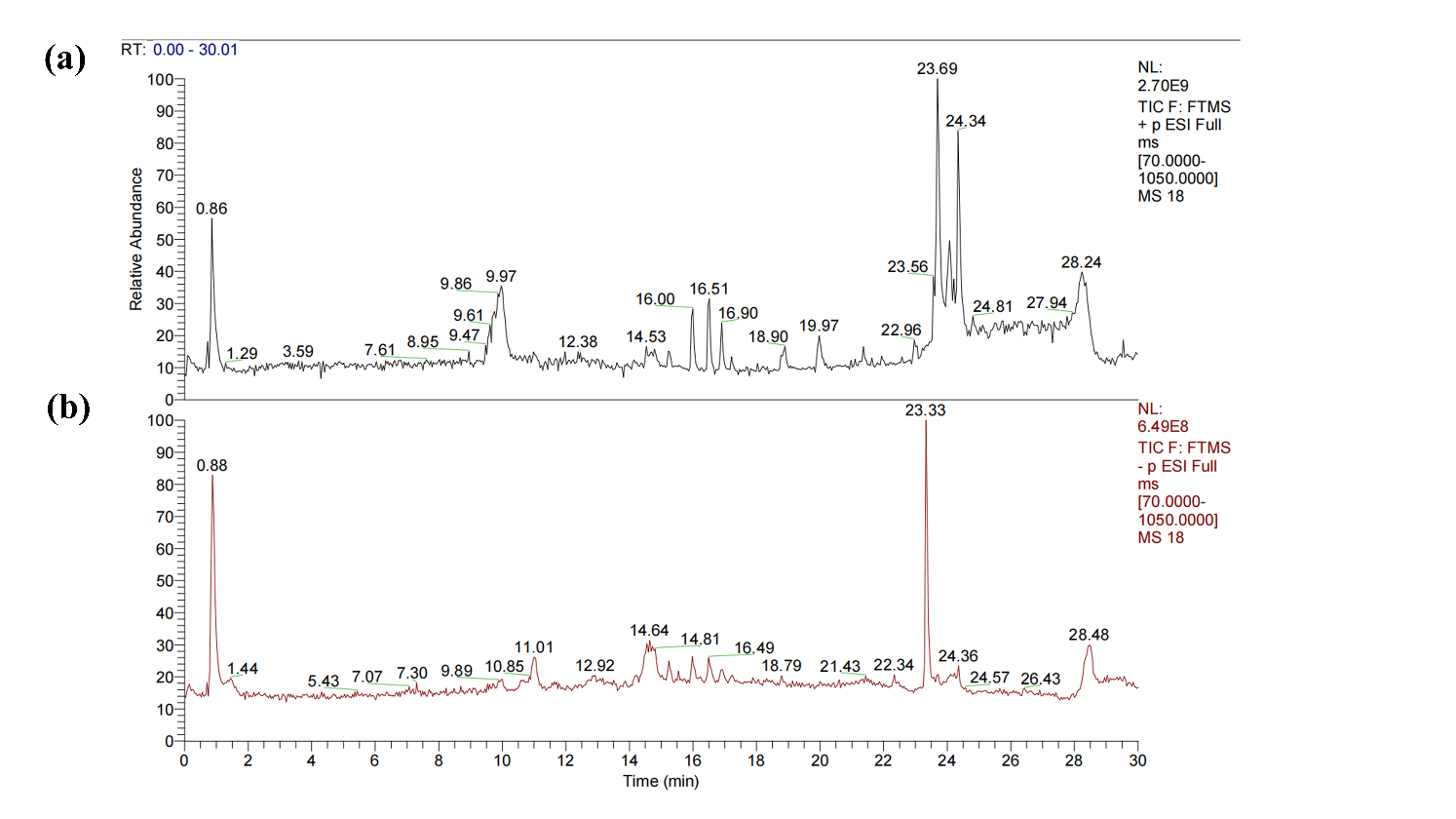


Figure S2 (a) Total ion chromatography of *Camellia sinensis var. assamica (Royle ex Hook.) (Theaceae)* (CS) extract in positive mode, (b) Total ion chromatography of CS extract in negative mode


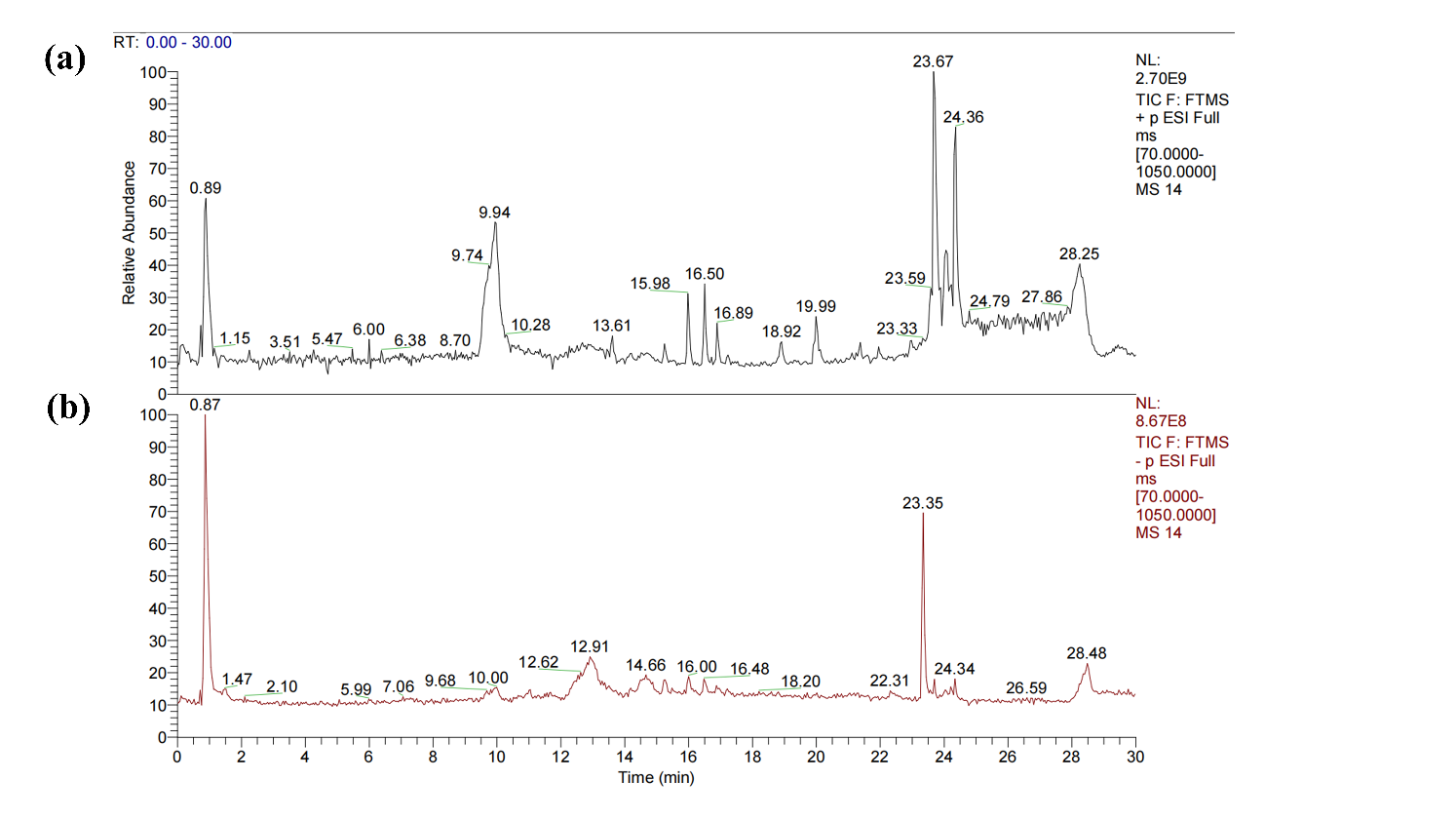


Figure S3 (a) Total ion chromatography of *Camellia ptilophylla Hung T. Chang (Theaceae)* (CP) extract in positive mode, (b) Total ion chromatography of CP extract in negative mode
